# Helicene synthesis by Brønsted acid-catalyzed cycloaromatization in HFIP [(CF_3_)_2_CHOH]

**DOI:** 10.3762/bjoc.17.35

**Published:** 2021-02-09

**Authors:** Takeshi Fujita, Noriaki Shoji, Nao Yoshikawa, Junji Ichikawa

**Affiliations:** 1 Division of Chemistry, Faculty of Pure and Applied Sciences, University of Tsukuba, Tsukuba, Ibaraki 305-8571, Japan

**Keywords:** acid, catalyst, cyclization, fluoroalcohol, helicenes

## Abstract

A facile synthesis of carbo- and heterohelicenes was achieved via tandem cycloaromatization of bisacetal precursors, which were readily prepared through C–C bond formation by Suzuki–Miyaura coupling. This cyclization was efficiently realized by a catalytic amount of trifluoromethanesulfonic acid (TfOH) in a cation-stabilizing solvent, 1,1,1,3,3,3-hexafluoropropan-2-ol (HFIP), which readily allowed gram-scale syntheses of higher-order helicenes, double helical helicenes, and heterohelicenes.

## Introduction

Helicenes are a class of polycyclic aromatic hydrocarbons (PAH) that consist of *ortho*-fused aromatic rings arranged in a helical manner [1–6]. Since helicenes possess chirality derived from their helical structure, optically active helicenes and their derivatives exhibit characteristic properties, such as huge optical rotation [7], nonlinear optical effects [8–10], and circularly polarized light emission [11], which are rarely found in planar aromatic hydrocarbons (e.g., acenes and phenacenes). Therefore, the wide breadth of applications of helicenes as organic optical materials make them interesting synthetic targets.

**Scheme 1:**
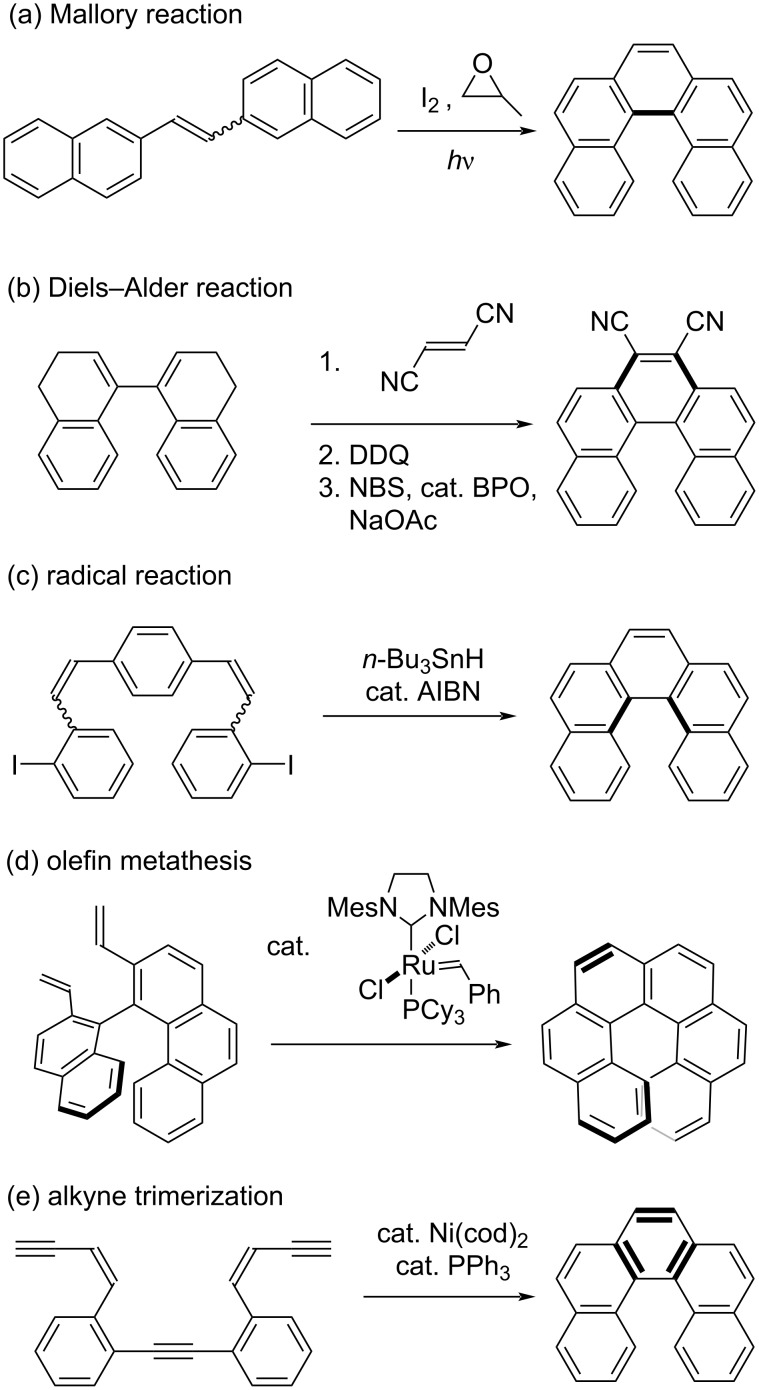
Conventional methods for the synthesis of helicenes.

Although several methods for the synthesis of helicenes have been reported, there are still specific problems in conducting these synthetic reactions for large scale production (Scheme 1). For example, the Mallory reaction [12], which is a widely used photocyclization in helicene synthesis [13–14], requires high dilution or excess amounts of oxidizing agents to suppress intermolecular side reactions (Scheme 1a). Diels–Alder (Scheme 1b) [15] and radical reactions (Scheme 1c) [16] directed toward helicene synthesis require high temperature conditions even for low to moderate yields. Olefin metathesis (Scheme 1d) [17] and alkyne trimerization (Scheme 1e) [18–19] require the use of expensive metal catalysts.

**Scheme 2:**
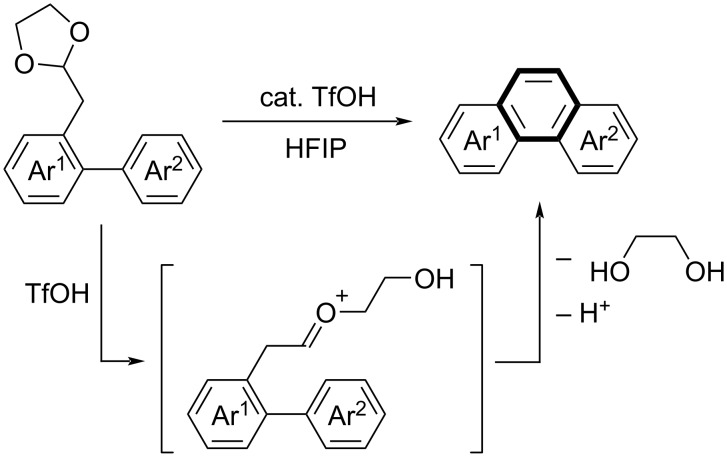
Brønsted acid-catalyzed cycloaromatization of biaryls bearing an acetal moiety.

Recently, we have developed a Brønsted acid-catalyzed cycloaromatization in 1,1,1,3,3,3-hexafluoropropan-2-ol (HFIP) as solvent [20–22]. Fluoroalcohols, such as HFIP, exhibit high ionization power and low nucleophilicity, based on the electron-withdrawing inductive effect of fluorine, which helps to generate cations but does not affect cationic reactions [23–27]. Thus, HFIP greatly facilitates reactions via cationic intermediates [28]. In the presence of a catalytic amount of trifluoromethanesulfonic acid in HFIP, (biaryl-2-yl)acetoaldehydes or their acetal derivatives readily underwent intramolecular Friedel–Crafts-type C–C bond formation followed by dehydration or alcohol elimination, leading to the construction of benzene rings in the biaryl systems (Scheme 2) [20–21]. The reaction proceeded via oxocarbenium ion intermediates stabilized by HFIP. This method can be successfully applied to cyclization at multiple reaction sites. By using bisacetals bearing a naphthalene core (Ar^2^) as substrates, tandem cyclization achieved the efficient synthesis of several *ortho*-fused six-hexagon benzenoids [21].

**Scheme 3:**
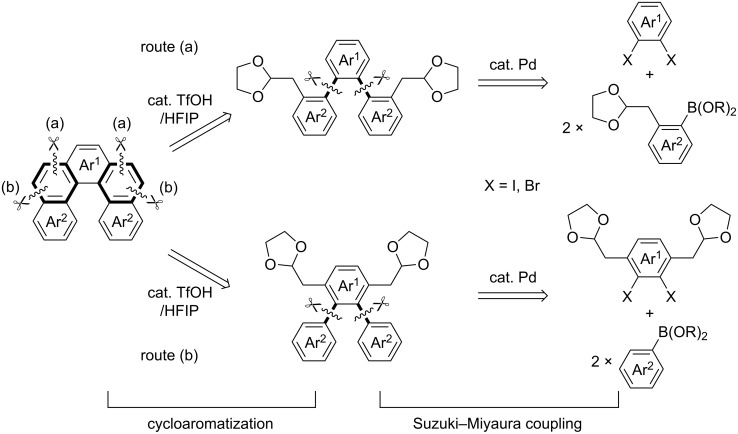
Two strategies for the helicene synthesis via Suzuki–Miyaura coupling/cycloaromatization sequence.

On the basis of our work mentioned above, we assumed that this powerful cycloaromatization would be applicable to helicene synthesis. Thus, the following strategies were designed to establish a versatile method for the synthesis of helicenes (Scheme 3). Tandem cycloaromatization of bisacetals bearing a teraryl core was shown to be most effective for the one-shot construction of helicene frameworks. Based on ease of preparation, symmetrical cyclization precursors would be preferable, and thus they should possess either (i) one acetal moiety on each terminal aromatic ring (Ar^2^) of the teraryl core (Scheme 3, route a) or (ii) two acetal moieties on the central aromatic ring (Ar^1^) (Scheme 3, route b). The teraryl structures were constructed by the formation of two C–C bonds via tandem Suzuki–Miyaura coupling of two terminal (Ar^2^) and central (Ar^1^) arenes. It is noted that dihalogenated arenes were adopted as components for the central aromatic ring (Ar^1^) in the teraryl structure, because the diborylated arenes were less available. Either (a) the coupling of boronic acid esters bearing one acetal moiety with dihalogenated arenes or (b) the coupling of dihalogenated arenes bearing two acetal moieties with arylboronic acids were conducted for the preparation of cyclization precursors. As described above, the tandem cycloaromatization of the obtained precursors proceeded via Friedel–Crafts-type bond formation followed by elimination, which enabled a rapid and efficient synthesis of helicenes, such as higher-order helicenes, double helical helicenes, and heterohelicenes.

## Results and Discussion

**Scheme 4:**
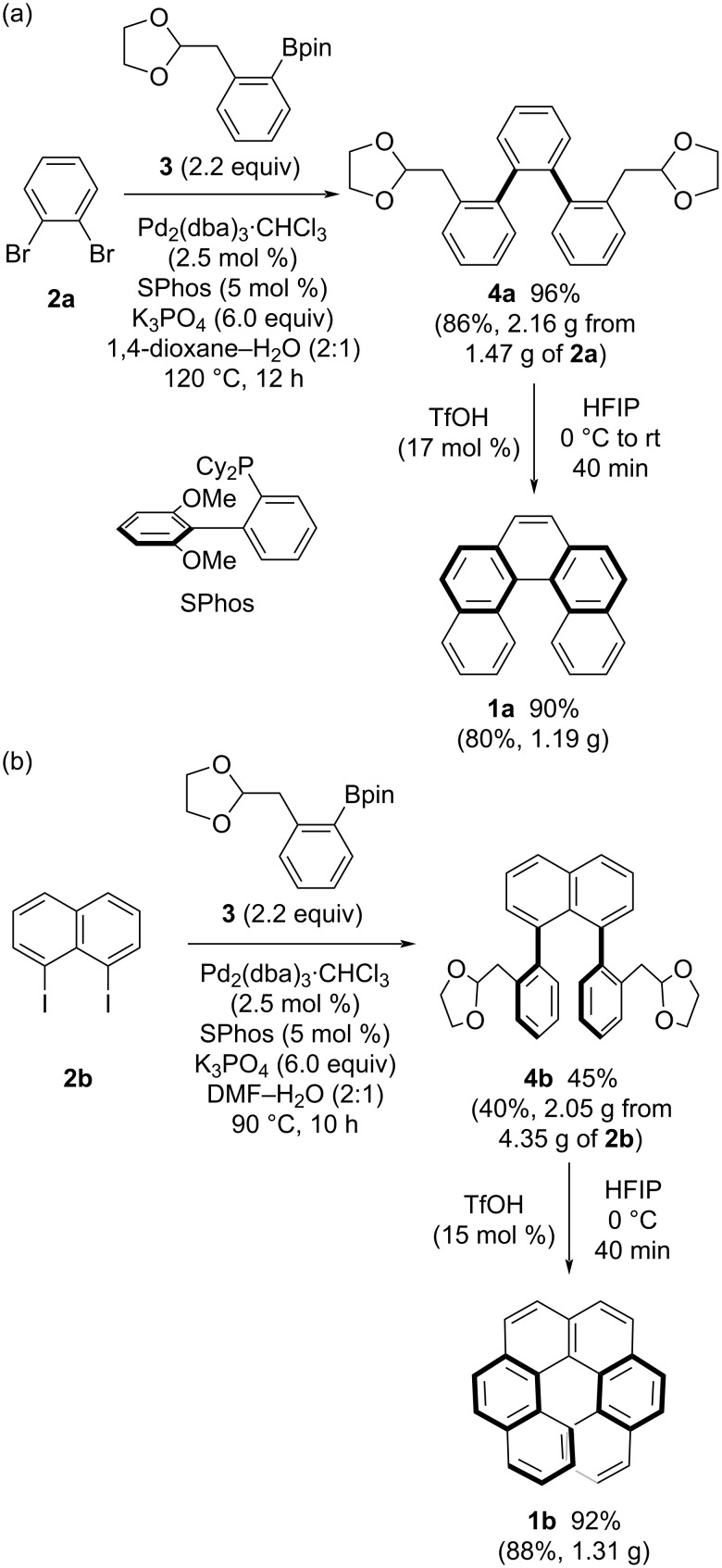
Synthesis of (a) [5]helicene and (b) [6]helicene.

Carbohelicenes such as [5]helicene (**1a**) and [6]helicene (**1b**) were synthesized via Suzuki–Miyaura coupling of readily available dihalogenated arenes **2** with phenylboronic acid ester **3** bearing a (1,3-dioxolan-2-yl)methyl group [29], followed by triflic acid-catalyzed cycloaromatization (Scheme 3, route a and Scheme 4a). Treatment of 1,2-dibromobenzene (**2a**) with **3** in the presence of a palladium catalyst with SPhos afforded *o*-terphenyl derivative **4a** bearing two acetal groups in 96% yield (Scheme 4a). The obtained bisacetal **4a** successfully underwent cycloaromatization in the presence of 17 mol % of TfOH in HFIP to afford [5]helicene (**1a**) in 90% yield. For the synthesis of [6]helicene, 1,8-diiodonaphthalene was adopted as the substrate for Suzuki–Miyaura coupling with **3** (Scheme 3, route a and Scheme 4b). In this coupling, SPhos was also effective in providing the bisacetal precursor **4b** bearing a 1,8-diphenylnaphthalene framework in 45% yield despite high steric hindrance (Scheme 4b). Then, [6]helicene (**1b**) was obtained in 92% yield via cycloaromatization in the presence of 15 mol % of TfOH. It was noted that the gram-scale synthesis for both [5]- and [6]helicenes was possible, in which no serious decrease in yield was observed in Suzuki–Miyaura coupling and cycloaromatization (see the Experimental section) [30].

**Scheme 5:**
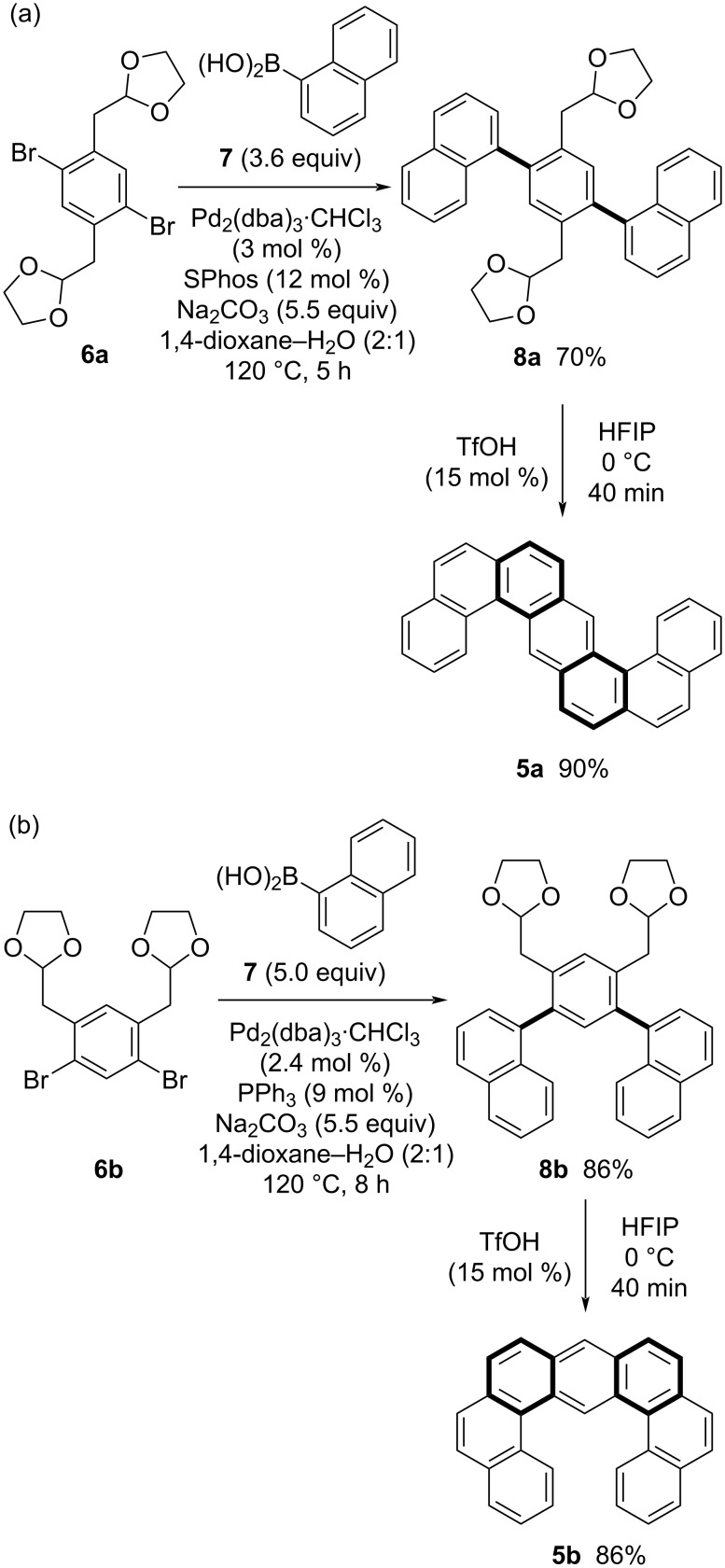
Synthesis of helicenes with double helical structures.

Next, double helical compounds were set as synthetic targets, because their unique optical properties have recently attracted much attention [5]. Herein, we synthesized two types of *ortho*-fused seven-hexagon benzenoids with two [4]helicene structures. According to the strategy illustrated in Scheme 3, route b, double helical helicenes **5** were synthesized using (naphthyl-1-yl)boronic acid (**7**) and dibromobenzenes **6** with two (1,3-dioxolan-2-yl)methyl groups, readily prepared from xylenes (Scheme 5). The Suzuki–Miyaura coupling of dibromobenzene **6a** with **7** effectively proceeded to afford bisacetal **8a** in 70% yield (Scheme 5a). Bisacetal **8a** successfully underwent subsequent cycloaromatization in HFIP to afford S-shaped double helical helicene **5a** in 90% yield. Similarly, C-shaped double helical helicene **5b** was obtained in high yield using *m*-dibromobenzene **6b** with two acetal moieties, prepared from *m*-xylene (Scheme 5b).

**Scheme 6:**
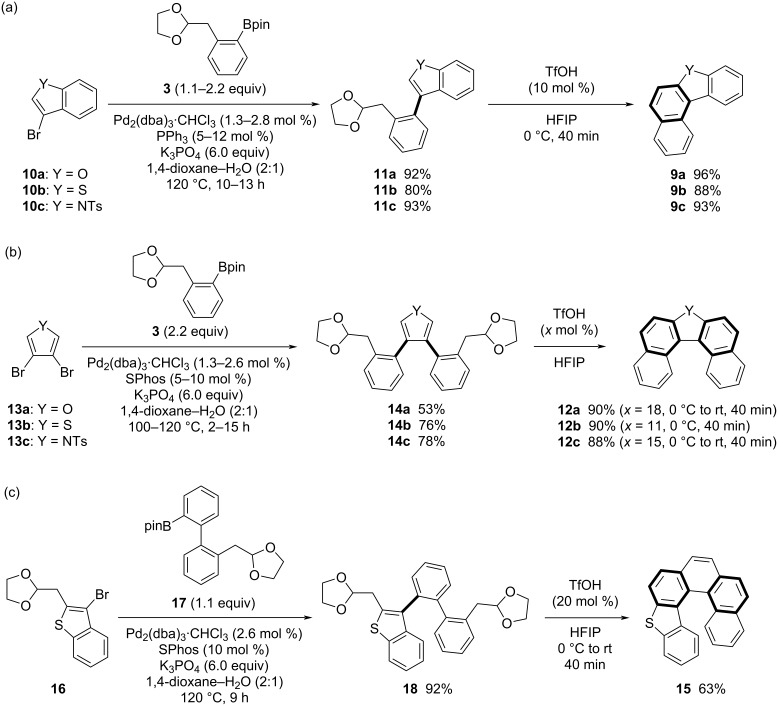
Synthesis of hetero[4]-, [5]-, and [6]helicenes.

We also achieved the synthesis of heterohelicenes, which involve heteroatoms in the helicene frameworks, via a Suzuki–Miyaura coupling/cycloaromatization sequence (Scheme 3a, route a). Thus, hetero[4]-, [5]-, and [6]helicenes involving an oxygen, sulfur, or nitrogen atom were efficiently synthesized (Scheme 6). Hetero[4]helicenes **9** were synthesized via coupling of 3-bromobenzoheteroles **10**, prepared from commercially available benzoheteroles, and subsequent cycloaromatization (Scheme 6a). Again, phenylboronic acid ester **3**, used above in the synthesis of [5]- and [6]helicenes, was adopted as the coupling partner for **10**. In the presence of a palladium catalyst, treatment of 3-bromobenzofuran (**10a**), 3-bromobenzothiophene (**10b**), and 3-bromo(*N*-tosyl)indole (**10c**) with **3** afforded the corresponding acetals **11a**–**c** with 3-phenylbenzoheterole structures in 92%, 80%, and 93% yields, respectively. Acetals **11a**–**c** successfully underwent TfOH-catalyzed cycloaromatization in HFIP to afford oxa-, thia-, and aza[4]helicenes **9a**–**c** in 96%, 88%, and 93% yields, respectively. Thus, phenylboronic acid ester **3** bearing an acetal moiety functions as a reagent for fused naphthalene ring extension through our cross-coupling/cycloaromatization sequence [31–35].

Hetero[5]helicenes **12** were synthesized via tandem cycloaromatization using teraryl precursors, in which heterol rings were set as central aromatic rings (Ar^1^) (Scheme 3, route a and Scheme 6b). As in the original strategy (Scheme 3, route a), coupling of 3,4-dibromofuran (**13a**), 3,4-dibromothiophene (**13b**), and 3,4-dibromo(*N*-tosyl)pyrrole (**13c**) with phenylboronic acid ester **3** bearing an acetal moiety proceeded to afford the corresponding bisacetals **14a**–**c** in 53%, 76%, and 78% yields, respectively. The obtained bisacetals **14a**–**c** efficiently underwent TfOH-catalyzed tandem cycloaromatization under appropriate conditions to afford oxa-, thia-, and aza[5]helicenes **12a**–**c** in 90%, 90%, and 88% yields, respectively.

Furthermore, we demonstrated the synthesis of unsymmetrical thia[6]helicene **15** as a hetero[6]helicene synthesis (Scheme 6c). 3-Bromobenzothiophene **16** bearing a (1,3-dioxolan-2-yl)methyl group at the 2-position, which was readily prepared from benzothiophene, underwent Suzuki–Miyaura coupling with boronic acid ester **17** with a biphenyl structure and an acetal moiety, to afford bisacetal **18** in 92% yield. Thus, tandem cycloaromatization of **18** effectively proceeded to afford thia[6]helicene **15** in 63% yield.

## Conclusion

In summary, we developed a facile and efficient method for the synthesis of several types of helicenes using a sequence of (i) Suzuki–Miyaura coupling of acetal-containing components and (ii) catalytic cycloaromatization with the aid of a cation-stabilizing fluoroalcohol, HFIP, as solvent. Particularly, helicene synthesis using tandem cyclization of bisacetals with teraryl structures is suitable for mass production of helicenes not only because multiple aromatic rings can be constructed at the same time but also because precursors can be easily prepared from commercially available compounds. Therefore, further progress can be made in applied research on helicenes as organic electronic materials, such as circularly polarized light emitting devices and nonlinear optical materials [7–11].

## Experimental

^1^H NMR and ^13^C NMR spectra were recorded on a Bruker Avance 500 or a JEOL ECS-400 spectrometer. Chemical shift values are given in ppm relative to internal Me_4_Si (for ^1^H NMR: δ = 0.00 ppm) and CDCl_3_ (for ^13^C NMR: δ = 77.0 ppm).

Column chromatography and preparative thin-layer chromatography (PTLC) were conducted on silica gel (Silica Gel 60 N, Kanto Chemical Co., Inc. for column chromatography and Wakogel B-5F, Wako Pure Chemical Inductries for PTLC). *N*,*N*-Dimethylformamide (DMF) was purified by a solvent-purification system (GlassContour) equipped with columns of activated alumina and supported-copper catalyst (Q-5) before use. 1,1,1,3,3,3-Hexafluoropropan-2-ol (HFIP) was distilled from molecular sieves 4 Å and stored over activated molecular sieves 4 Å. 1,4-Dioxane was distilled from sodium and stored over activated molecular sieves 4 Å. 2-{2-[(1,3-Dioxolan-2-yl)methyl]phenyl}-4,4,5,5-tetramethyl-1,3,2-dioxaborolane (**3**) was prepared according to the literature procedure, and their spectral data showed good agreement with the literature data [21]. Unless otherwise noted, materials were obtained from commercial sources and used directly without further purification.

### 2,2''-Bis[(1,3-dioxolan-2-yl)methyl]-1,1':2',1''-terphenyl (**4a**)

A dioxane (6.6 mL) and H_2_O (3.4 mL) solution of 1,2-dibromobenzene (**2a**, 471 mg, 2.00 mmol), boronate ester **3** (1.27 g, 4.39 mmol), Pd_2_(dba)_3_·CHCl_3_ (52 mg, 51 μmol), SPhos (41 mg, 0.10 mmol), and K_3_PO_4_ (2.53 g, 11.9 mmol) was degassed by using the freeze-pump-thaw method three times. After stirring at 120 °C for 12 h, ethyl acetate and water were added to the mixture, and organic materials were extracted with ethyl acetate three times. The combined extracts were washed with brine and dried over Na_2_SO_4_. After removal of the solvent under reduced pressure, the residue was purified by silica gel column chromatography (hexane/EtOAc = 3:1) to give **4a** (776 mg, 96%) as an orange oil.

^1^H NMR (500 MHz, CDCl_3_) δ 2.59 (dd, *J* = 14.4, 4.9 Hz, 0.8H), 2.70 (dd, *J* = 14.3, 5.3 Hz, 1.2H), 2.78–2.86 (m, 2H), 3.72–3.95 (m, 8H), 4.85–4.95 (m, 2H), 6.97–7.03 (m, 4H), 7.07–7.14 (m, 2H), 7.23–7.33 (m, 3.2H), 7.35–7.42 (m, 2.8H); ^13^C NMR (126 MHz, CDCl_3_) δ 37.1, 37.7, 64.6, 64.73, 64.75, 104.5, 104.6, 125.4, 125.7, 126.8, 126.87, 126.91, 126.0, 129.5, 129.6, 130.0, 130.7, 131.2, 131.6, 133.8, 134.0, 140.2, 140.9, 141.4; IR (neat) ν: 3055, 3020, 2976, 2883, 1471, 1442, 1433, 1398, 1194, 1130, 1038, 1009, 943, 872, 837, 822, 752, 706, 621, 573, 538 cm^−1^; HRMS (EI) *m*/*z*: [M]^+^ calcd. for C_26_H_26_O_4_, 402.1826; found, 402.1815.

**Gram-scale synthesis:** Compound **4a** was also prepared by the method described above using 1,2-dibromobenzene (**2a**, 1.47 g, 6.24 mmol), boronate ester **3** (3.99 g, 13.8 mmol), Pd_2_(dba)_3_·CHCl_3_ (82 mg, 80 μmol), PPh_3_ (84 mg, 0.32 mmol), K_3_PO_4_ (8.02 g, 37.8 mmol), dioxane (21 mL), and H_2_O (10 mL) at 100 ºC for 6 h. Purification by silica gel column chromatography (hexane/EtOAc = 5:1) gave **4a** (2.16 g, 86%).

### [5]Helicene (**1a**)

In a similar manner as described in [20], trifluoromethanesulfonic acid (7.5 mg, 50 μmol) was added to an HFIP (0.9 mL) solution of bisacetal **4a** (120 mg, 0.30 mmol) at 0 °C. After stirring at room temperature for 40 min, the reaction was quenched with phosphate buffer (pH 7). Organic materials were extracted with CH_2_Cl_2_ three times, and the combined extracts were washed with brine and dried over Na_2_SO_4_. After removal of the solvents under reduced pressure, the residue was purified by silica gel column chromatography (hexane/CH_2_Cl_2_ = 3:1) to give **1a** (75 mg, 90%) as a white solid.

^1^H NMR (500 MHz, CDCl_3_) δ 7.23 (dd, *J* = 8.4, 7.1 Hz, 2H), 7.47 (dd, *J* = 7.9, 7.1 Hz, 2H), 7.80 (s, 2H), 7.81 (d, *J* = 8.5 Hz, 2H), 7.86 (d, *J* = 8.5 Hz, 2H), 7.90 (d, *J* = 7.9 Hz, 2H), 8.49 (d, *J* = 8.4 Hz, 2H); ^13^C NMR (126 MHz, CDCl_3_) δ 124.3, 126.2, 126.3, 126.9, 127.2, 127.4, 127.8, 129.0, 130.7, 132.2, 132.5.

**Gram-scale synthesis:** Compound **1a** was also synthesized by the method described above using bisacetal **4a** (2.16 g, 5.37 mmol), trifluoromethanesulfonic acid (88 mg, 0.58 mmol), and HFIP (20 mL) at 0 °C for 40 min. Purification by silica gel column chromatography (hexane/EtOAc = 10:1) gave **1a** (1.19 g, 80%).

### 1,8-Bis{2-[(1,3-dioxolan-2-yl)methyl]phenyl}naphthalene (**4b**)

A DMF (6.7 mL) and H_2_O (3.3 mL) solution of 1,8-diiodonaphthalene (**2b**, 759 mg, 2.00 mmol), boronate ester **3** (1.28 g, 4.40 mmol), Pd_2_(dba)_3_·CHCl_3_ (52 mg, 50 μmol), SPhos (42 mg, 0.10 mmol), and K_3_PO_4_ (2.54 g, 12.0 mmol) was degassed by using the freeze-pump-thaw method three times. After stirring at 90 °C for 10 h, ethyl acetate, hexane, and water were added to the mixture, and organic materials were extracted with an ethyl acetate–hexane (1:1) mixed solvent three times. The combined extracts were washed with brine and dried over Na_2_SO_4_. After removal of the solvent under reduced pressure, the residue was purified by silica gel column chromatography (toluene/EtOAc = 40:1) to give **4b** (409 mg, 45%) as a white solid.

^1^H NMR (500 MHz, CDCl_3_) δ 2.36 (dd, *J* = 14.0, 6.1 Hz, 2H), 2.52 (dd, *J* = 14.0, 4.3 Hz, 2H), 3.62–3.83 (m, 8H), 4.65 (dd, *J* = 6.1, 4.3 Hz, 2H), 6.77–6.81 (m, 2H), 6.87–6.93 (m, 4H), 7.01–7.06 (m, 2H), 7.20 (dd, *J* = 7.0, 1.3 Hz, 2H), 7.46 (dd, *J* = 8.1, 7.0 Hz, 2H), 7.91 (dd, *J* = 8.3, 1.3 Hz, 2H); ^13^C NMR (126 MHz, CDCl_3_) δ 38.9, 64.4, 64.7, 104.1, 124.8, 125.2, 126.8, 129.0, 129.05, 129.13, 129.9, 131.0, 133.8, 134.5, 138.8, 142.6; IR (neat) ν: 3053, 2966, 2883, 1489, 1396, 1132, 1043, 984, 831, 756 cm^–1^; HRMS (APCI+) *m*/*z*: [M + H]^+^ calcd. for C_30_H_29_O_4_, 453.2060; found, 453.2081.

**Gram-scale synthesis:** Compound **4b** was also prepared by the method described above using 1,8-diiodonaphthalene (**2b**, 4.35 g, 11.4 mmol), boronate ester **3** (7.31 g, 25.2 mmol), Pd_2_(dba)_3_·CHCl_3_ (301 mg, 0.29 mmol), SPhos (239 mg, 0.58 mmol), K_3_PO_4_ (14.6 g, 68.7 mmol), DMF (38 mL), and H_2_O (19 mL) at 90 °C for 17 h. Purification by silica gel column chromatography (toluene/EtOAc = 40:1) gave **4b** (2.05 g, 40%).

### [6]Helicene (**1b**)

In a similar manner as described in [20]. To an HFIP (0.7 mL) solution of bisacetal **4b** (91 mg, 0.20 mmol) was added trifluoromethanesulfonic acid (4.8 mg, 32 μmol) at 0 °C. After stirring at the same temperature for 40 min, the reaction was quenched with phosphate buffer (pH 7). Organic materials were extracted with ethyl acetate three times, and the combined extracts were washed with brine and dried over Na_2_SO_4_. After removal of the solvents under reduced pressure, the residue was purified by silica gel column chromatography (hexane/CH_2_Cl_2_ = 3:1) to give **1b** (60 mg, 92%) as a yellow solid.

^1^H NMR (500 MHz, CDCl_3_) δ 6.66 (dd, *J* = 8.5, 7.4 Hz, 2H), 7.19 (dd, *J* = 7.9, 7.4 Hz, 2H), 7.58 (d, *J* = 8.5 Hz, 2H), 7.80 (d, *J* = 7.9 Hz, 2H), 7.89 (s, 4H), 7.93 (d, *J* = 8.1 Hz, 2H), 7.96 (d, *J* = 8.1 Hz, 2H); ^13^C NMR (126 MHz, CDCl_3_) δ 124.0, 124.6, 125.5, 126.1, 126.8, 127.2, 127.5, 127.7, 127.8, 128.0, 129.9, 131.2, 131.7, 133.1.

**Gram-scale synthesis:** Compound **1b** was also synthesized by the method described above using bisacetal **4b** (2.05 g, 4.53 mmol), trifluoromethanesulfonic acid (173 mg, 1.2 mmol), and HFIP (15 mL) at 0 ºC for 3 h. Purification by silica gel column chromatography (hexane/CH_2_Cl_2_ = 3:1) gave **1b** (1.31 g, 88%).

## Supporting Information

File 1 Detailed experimental procedures and spectral data.
