# Supplementary material for: Helicene synthesis by Brønsted acid-catalyzed cycloaromatization in HFIP [(CF3)2CHOH]
Source: Beilstein J Org Chem. 2021 Feb 9;17:396–403. doi: 10.3762/bjoc.17.35 (PMC7884880; doi:10.3762/bjoc.17.35)

BEILSTEIN JOURNAL OF ORGANIC CHEMISTRY

Supporting Information

for

Helicene synthesis by Brønsted acid-catalyzed cycloaromatization in HFIP [$(CF_3)_2CHOH$]

Takeshi Fujita, Noriaki Shoji, Nao Yoshikawa and Junji Ichikawa

Beilstein J. Org. Chem. **2021,** *17,* 396–403. doi:10.3762/bjoc.17.35

Detailed experimental procedures and spectral data

License and Terms: This is a supporting information file under the terms of the Creative Commons Attribution License (https://creativecommons.org/licenses/by/4.0). Please note that the reuse, redistribution and reproduction in particular requires that the author(s) and source are credited and that individual graphics may be subject to special legal provisions.
The license is subject to the *Beilstein Journal of Organic Chemistry* terms and conditions: (https://www.beilstein-journals.org/bjoc/terms)

1. General statement S2

2. Preparation of cyclization precursors S3

3. Synthesis of helicenes S14

4. References S19

5. ^{1}H and ^{13}C NMR charts S21

1. General statement

^{1}H NMR and ^{13}C NMR spectra were recorded on a Bruker Avance 500 or a JEOL ECS-400 spectrometer. Chemical shift values are given in ppm relative to internal Me_4Si (for ^{1}H NMR: δ = 0.00 ppm) and $CDCl_3$ (for ^{13}C NMR: δ = 77.0 ppm). IR spectra were recorded on a Horiba FT-300S spectrometer by the attenuated total reflectance (ATR) method. Mass spectra were measured on a JEOL JMS-T100GCV or a JEOL JMS-T100CS spectrometer. Elemental analyses were carried out at the Elemental Analysis Laboratory, Division of Chemistry, Faculty of Pure and Applied Sciences, University of Tsukuba.

Column chromatography and preparative thin-layer chromatography (PTLC) were conducted on silica gel (Silica Gel 60 N, Kanto Chemical Co., Inc. for column chromatography and Wakogel B-5F, Wako Pure Chemical Inductries for PTLC). *N*,*N*-dimethylformamide (DMF) was purified by a solvent-purification system (GlassContour) equipped with columns of activated alumina and supported-copper catalyst (Q-5) before use. 1,1,1,3,3,3-Hexafluoropropan-2-ol (HFIP) was distilled from molecular sieves 4 Å and stored over activated molecular sieves 4 Å. 1,4-Dioxane was distilled from sodium and stored over activated molecular sieves 4 Å. 1,8-Diiodonaphthalene (**2b**) [1], 2-{2-[(1,3-dioxolan-2-yl)methyl]phenyl}-4,4,5,5-tetramethyl-1,3,2-dioxaborolane (**3**) [2], 2,5-dibromoterephthalaldehyde [3], 4,6-dibromoisophthalaldehyde [4], 3-bromobenzofuran (**10a**) [5], 3-bromobenzo[*b*]thiophene (**10b**) [6], 3-bromo-1-(4-methylbenzenesulfonyl)-1*H*-indole (**10c**) [7], 3,4-dibromofuran (**13a**) [8], 3,4-dibromothiophene (**13b**) [9], 3,4-dibromo-1-(4-methylbenzenesulfonyl)-1*H*-pyrrole (**13c**) [10], and 3-bromobenzo[*b*]thiophene-2-carbaldehyde [5] were prepared according to the literature procedures, and their spectral data showed good agreement with the literature data. Unless otherwise noted,

materials were obtained from commercial sources and used directly without further purifications.

2. Preparation of cylization precursors

2,2'-{[2,5-Di(naphthalen-1-yl)-1,4-phenylene]bis(methylene)}bis(1,3-dioxolane) (8a)

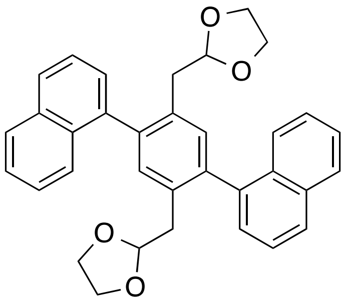

A dioxane (0.66 mL) and H_2O (0.34 mL) solution of dibromobisacetal **6a** (92 mg, 0.22 mmol), (naphthalen-1-yl)boronic acid (**7**, 137 mg, 0.80 mmol), $Pd_2(dba)_3{\cdot}CHCl_3$ (6.7 mg, 6.5 μmol), SPhos (11 mg, 27 μmol), and Na_2CO_3 (129 mg, 1.2 mmol) was degassed by using the freeze-pump-thaw method three times. After stirring at 120 °C for 5 h, ethyl acetate and water were added to the mixture, and organic materials were extracted with ethyl acetate three times. The combined extracts were washed with brine and dried over Na_2SO_4. After removal of the solvent under reduced pressure, the residue was purified by silica gel column chromatography (toluene/EtOAc = 30:1) to give **8a** (78 mg, 70%) as a white solid.

^{1}H NMR (500 MHz, $CDCl_3$): δ 2.64–2.82 (m, 4H), 3.60–3.75 (m, 8H), 4.86–4.90 (m, 2H), 7.39–7.60 (m, 10H), 7.63–7.69 (m, 2H), 7.86–7.96 (m, 4H). ^{13}C NMR (126 MHz, $CDCl_3$): δ 37.49, 37.53, 64.53, 64.55, 104.2, 104.3, 125.3, 125.69, 125.74, 125.9, 126.0, 126.3, 126.4, 127.4, 127.59, 127.61, 128.1, 128.2, 132.38, 132.42, 132.47, 132.51, 133.0, 133.1, 133.6, 139.00, 139.03, 139.7, 139.8. IR (neat): ν 3057, 2964,

2883, 1508, 1396, 1128, 1045, 804, 777, 669 cm^{-1}. HRMS (APCI+): *m/z* Calcd. for $C_{34}H_{31}O_4$ [M + H]$^+$: 503.2217; Found: 503.2204.

2,2'-{[4,6-Di(naphthalen-1-yl)-1,3-phenylene]bis(methylene)}bis(1,3-dioxolane) (8b)

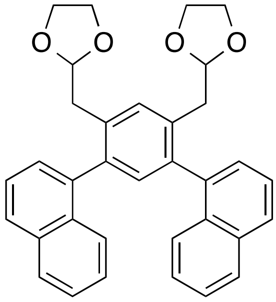

Compound **8b** was also prepared by the method described for compound **8a** using dibromobisacetal **6b** (490 mg, 1.20 mmol), (naphthalen-1-yl)boronic acid (**7**, 1.03 g, 6.00 mmol), $Pd_2(dba)_3{\cdot}CHCl_3$ (30 mg, 29 μmol), PPh_3 (30 mg, 0.11 mmol), Na_2CO_3 (704 mg, 6.64 mmol), dioxane (4.0 mL), and H_2O (2.0 mL) at 120 ºC for 8 h. Purification by silica gel column chromatography (hexane/EtOAc = 3:1) gave **8b** (517 mg, 86%) as a white solid.

^{1}H NMR (500 MHz, $CDCl_3$): δ 2.74–2.78 (m, 2H), 2.86–2.89 (m, 2H), 3.71–3.84 (m, 8H), 4.96–4.98 (m, 2H), 7.16–7.17 (m, 1H), 7.38–7.50 (m, 8H), 7.61–7.66 (m, 3H), 7.82 (d, *J* = 8.1 Hz, 2H), 7.85 (d, *J* = 8.2 Hz, 2H). ^{13}C NMR (126 MHz, $CDCl_3$): δ 37.8, 37.9, 64.6, 64.7, 104.36, 104.39, 125.1, 125.62, 125.64, 125.89, 125.93, 126.2, 126.3, 127.3, 127.4, 127.6, 128.1, 131.9, 132.1, 132.3, 132.4, 132.9, 133.1, 133.5, 134.2, 134.3, 138.62, 138.63, 138.7, 138.8. IR (neat): ν 3057, 2968, 2883, 1508, 1394, 1130, 1039, 945, 779, 669 cm^{-1}. HRMS (APCI+): *m/z* Calcd. for $C_{34}H_{31}O_4$ [M + H]$^+$: 503.2217; Found: 503.2234.

3-{2-[(1,3-Dioxolan-2-yl)methyl]phenyl}benzofuran (11a)

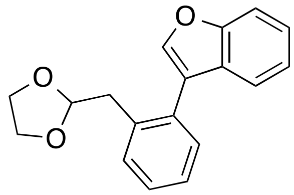

A dioxane (3.3 mL) and H_2O (1.7 mL) solution of 3-bromobenzofuran (**10a**, 197 mg, 1.0 mmol), boronate ester **3** (319 mg, 1.10 mmol), $Pd_2(dba)_3\cdot CHCl_3$ (13 mg, 13 μmol), PPh_3 (13 mg, 50 μmol), and K_3PO_4 (1.27 g, 5.98 mmol) was degassed by using the freeze-pump-thaw method three times. After stirring at 120 °C for 13 h, ethyl acetate and water were added to the mixture, and organic materials were extracted with ethyl acetate three times. The combined extracts were washed with brine and dried over Na_2SO_4. After removal of the solvent under reduced pressure, the residue was purified by silica gel column chromatography (hexane/EtOAc = 10/1 and hexane/EtOAc = 20:1) to give **11a** (258 mg, 92%) as a yellow solid.

^{1}H NMR (500 MHz, $CDCl_3$): δ 3.03 (d, J = 4.9 Hz, 2H), 3.69–3.94 (m, 4H), 5.00 (t, J = 4.9 Hz, 1H), 7.20–7.25 (m, 1H), 7.29–7.38 (m, 3H), 7.40 (d, J = 7.3 Hz, 1H), 7.43 (d, J = 7.8 Hz, 1H), 7.50 (d, J = 7.1 Hz, 1H), 7.53 (d, J = 8.3 Hz, 1H), 7.74 (s, 1H). ^{13}C NMR (126 MHz, $CDCl_3$): δ 37.7, 64.8, 104.5, 111.5, 120.3, 120.7, 122.8, 124.4, 126.7, 127.8, 128.1, 130.76, 130.82, 131.4, 135.1, 142.6, 155.0. IR (neat): ν 3064, 2956, 2883, 1606, 1489, 1473, 1452, 1398, 1333, 1219, 1132, 1109, 1086, 1036, 991, 964, 943, 858, 746 cm^{-1}. HRMS (EI) *m*/*z* Calcd. for $C_{18}H_{16}O_3$ $[M]^+$: 280.1094; Found: 280.1108.

2-[2-(Benzo[*b*]thiophen-3-yl)benzyl]-1,3-dioxolane (11b)

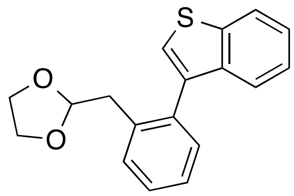

Compound **11b** was prepared by the method described for compound **11a** using 3-bromobenzo[*b*]thiophene (**10b**, 82 mg, 0.39 mmol), boronate ester **3** (125 mg, 0.43 mmol), $Pd_2(dba)_3{\cdot}CHCl_3$ (11 mg, 11 μmol), PPh_3 (12 mg, 46 μmol), K_3PO_4 (486 mg, 2.29 mmol), dioxane (1.3 mL), and H_2O (0.6 mL) at 120 ºC for 13 h. Purification by silica gel column chromatography (hexane/EtOAc = 10:1 and hexane/CH_2Cl_2 = 1:2) gave **11b** (91 mg, 80%) as a yellow solid.

^{1}H NMR (500 MHz, $CDCl_3$): δ 2.87 (d, *J* = 5.0 Hz, 2H), 3.62–3.85 (m, 4H), 4.92 (t, *J* = 5.0 Hz, 1H), 7.26–7.43 (m, 7H), 7.50 (d, *J* = 7.6 Hz, 1H), 7.87 (d, *J* = 7.8 Hz, 1H). ^{13}C NMR (126 MHz, $CDCl_3$): δ 37.7, 64.6, 104.3, 122.6, 123.0, 124.1, 124.2, 124.3, 126.5, 127.8, 130.6, 130.7, 135.3, 135.8, 136.7, 139.3, 139.8. IR (neat): ν 3060, 3022, 2962, 2881, 1483, 1425, 1396, 1340, 1257, 1214, 1190, 1128, 1103, 1036, 987, 943, 840, 813, 762, 734, 638, 534, 509 cm^{-1}. HRMS (EI) *m*/*z* Calcd. for $C_{18}H_{16}O_2S$ [M]$^+$: 296.0866; Found: 296.0878.

3-{2-[(1,3-Dioxolan-2-yl)methyl]phenyl}-1-(4-methylbenzenesulfonyl)-1*H*-indole (11c)

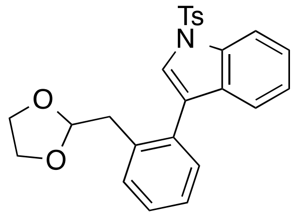

Compound **11c** was prepared by the method described for compound **11a** using 3-bromo-1-(4-methylbenzenesulfonyl)-1*H*-indole (**10c**, 526 mg, 1.50 mmol), boronate ester **3** (958 mg, 3.30 mmol), $Pd_2(dba)_3{\cdot}CHCl_3$ (20 mg, 19 μmol), PPh_3 (20 mg, 76 μmol), K_3PO_4 (1.92 g, 9.04 mmol), dioxane (5.0 mL), and H_2O (2.5 mL) at 120 °C for 10 h. Purification by silica gel column chromatography (hexane/EtOAc = 5:1 and hexane/CH_2Cl_2 = 1:2) gave **11c** (608 mg, 93%) as a yellow oil.

^{1}H NMR (500 MHz, $CDCl_3$): δ 2.28 (s, 3H), 2.91 (d, *J* = 5.0 Hz, 2H), 3.70–3.92 (m, 4H), 4.98 (t, *J* = 5.0 Hz, 1H), 7.15–7.21 (m, 3H), 7.25–7.35 (m, 5H), 7.47 (d, *J* = 7.8 Hz, 1H), 7.74 (s, 1H), 7.79 (d, *J* = 8.4 Hz, 2H), 8.05 (dd, *J* = 8.1, 1.1 Hz, 1H). ^{13}C NMR (126 MHz, $CDCl_3$): δ 21.4, 37.6, 64.7, 104.5, 113.6, 120.3, 122.6, 123.4, 124.7, 124.9, 126.5, 126.7, 127.8, 129.8, 130.71, 130.73, 131.0, 132.4, 134.8, 134.98, 135.05, 144.8. IR (neat): ν 3156, 3064, 3020, 2960, 1597, 1491, 1444, 1398, 1369, 1306, 1188, 1174, 1126, 1111, 1088, 1036, 1020, 1011, 931, 811, 742, 717, 665, 636, 600, 569, 536 cm^{-1}. HRMS (ESI+) *m*/*z* Calcd. for $C_{25}H_{23}NNaO_4S$ $[M + Na]^+$: 456.1240; Found: 456.1261.

3,4-Bis{2-[(1,3-dioxolan-2-yl)methyl]phenyl}furan (14a)

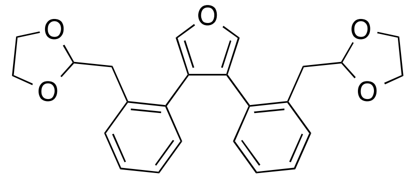

A dioxane (1.7 mL) and H_2O (0.8 mL) solution of 3,4-dibromofuran (**13a**, 113 mg, 0.50 mmol), boronate ester **3** (319 mg, 1.10 mmol), $Pd_2(dba)_3$·$CHCl_3$ (13 mg, 13 μmol), SPhos (10 mg, 25 μmol), and K_3PO_4 (642 mg, 3.02 mmol) was degassed by using the freeze-pump-thaw method three times. After stirring at 120 °C for 15 h, ethyl acetate and water were added to the mixture, and organic materials were extracted with ethyl acetate three times. The combined extracts were washed with brine and dried over Na_2SO_4. After removal of the solvent under reduced pressure, the residue was purified by silica gel column chromatography (hexane/EtOAc = 5:1) to give **14a** (104 mg, 53%) as a yellow oil.

^{1}H NMR (500 MHz, $CDCl_3$): δ 2.86 (d, *J* = 4.9 Hz, 4H), 3.72–3.94 (m, 8H), 4.78 (t, *J* = 4.9 Hz, 2H), 7.03–7.10 (m, 4H), 7.18 (ddd, *J* = 7.6, 7.5, 1.6 Hz, 2H), 7.31 (d, *J* = 7.6

Hz, 2H), 7.58 (s, 2H). ^{13}C NMR (126 MHz, $CDCl_3$): δ 37.4, 64.6, 104.4, 125.4, 126.2, 127.3, 130.4, 131.1, 131.8, 134.8, 141.2. IR (neat): ν 3060, 2956, 2883, 1761, 1734, 1558, 1543, 1506, 1489, 1473, 1456, 1446, 1398, 1362, 1338, 1223, 1134, 1036, 991, 954, 945, 877, 822, 764, 706, 611, 598, 573 cm^{-1}. HRMS (ESI+) *m*/*z* Calcd. for $C_{24}H_{24}NaO_5$ [M + Na]$^+$: 415.1516; Found: 415.1502.

3,4-Bis{2-[(1,3-dioxolan-2-yl)methyl]phenyl}thiophene (14b)

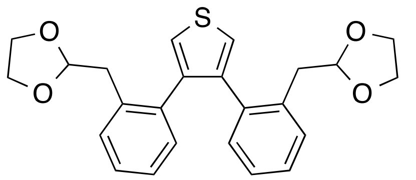

Compound **14b** was prepared by the method described for compound **14a** using 3,4-dibromothiophene (**13b**, 411 mg, 1.70 mmol), boronate ester **3** (1.08 g, 3.74 mmol), $Pd_2(dba)_3{\cdot}CHCl_3$ (22 mg, 21 μmol), SPhos (38 mg, 93 μmol), K_3PO_4 (2.17 g, 10.2 mmol), dioxane (5.6 mL), and H_2O (2.8 mL) at 100 °C for 2 h. Purification by silica gel column chromatography (hexane) gave **14b** (530 mg, 76%) as a brown solid.

^{1}H NMR (500 MHz, $CDCl_3$): δ 2.81 (d, *J* = 5.0 Hz, 4H), 3.76–3.91 (m, 8H), 4.81 (t, *J* = 5.0 Hz, 2H), 7.02–7.06 (m, 4H), 7.15 (dd, J = 7.7, 7.7 Hz, 2H), 7.29 (d, J = 7.7 Hz, 2H), 7.34 (s, 2H). ^{13}C NMR (126 MHz, $CDCl_3$): δ 37.3, 64.7, 104.5, 124.4, 125.9, 127.2, 130.0, 131.0, 134.6, 136.5, 141.4. IR (neat): ν 3060, 2883, 1481, 1396, 1132, 1035, 822, 760, 580 cm^{-1}. Elem. Anal. Cald. for $C_{24}H_{24}O_4S$: C 70.56%, H 5.92%; Found: C 70.20%, H 5.76%.

3,4-Bis{2-[(1,3-dioxolan-2-yl)methyl]phenyl}-1-(4-methylbenzenesulfonyl)-1*H*-pyrrole (14c)

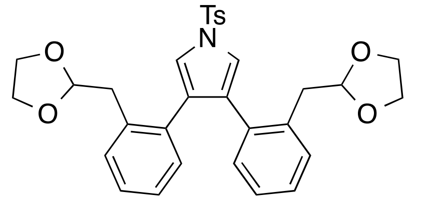

Compound **14c** was prepared by the method described for compound **14a** using 3,4-dibromo-1-(4-methylbenzenesulfonyl)-1*H*-pyrrole (**13c**, 113 mg, 0.30 mmol), boronate ester **3** (192 mg, 0.66 mmol), $Pd_2(dba)_3·CHCl_3$ (7.9 mg, 7.6 μmol), SPhos (12 mg, 29 μmol), K_3PO_4 (384 mg, 1.81 mmol), dioxane (1.0 mL), and H_2O (0.5 mL) at 120 °C for 5 h. Purification by silica gel column chromatography (hexane/EtOAc = 3:1 and 2:1) gave **14c** (128 mg, 78%) as a yellow solid.

^{1}H NMR (500 MHz, $CDCl_3$): δ 2.42 (s, 3H), 2.75 (d, *J* = 5.0 Hz, 4H), 3.68–3.91 (m, 8H), 4.72 (t, *J* = 5.0 Hz, 2H), 6.93 (d, *J* = 7.6 Hz, 2H), 7.01 (dd, *J* = 7.3, 7.3 Hz, 2H), 7.14 (ddd, *J* = 7.6, 7.3, 1.0 Hz, 2H), 7.26 (d, *J* = 7.3 Hz, 2H), 7.31 (d, *J* = 8.3 Hz, 2H), 7.33 (s, 2H), 7.81 (d, *J* = 8.3 Hz, 2H). ^{13}C NMR (126 MHz, $CDCl_3$): δ 21.6, 37.3, 64.6, 104.4, 120.1, 126.1, 126.8, 127.2, 128.6, 129.9, 130.2, 131.1, 133.3, 134.6, 136.0 144.9. IR (neat): ν 3136, 3060, 3020, 2960, 2885, 1597, 1529, 1475, 1442, 1400, 1371, 1324, 1188, 1173, 1132, 1092, 1076, 1053, 989, 951, 948, 874, 814, 754, 704, 671, 594, 540, 521 cm^{-1}. HRMS (ESI+) *m*/*z* Calcd. for $C_{31}H_{31}NNaO_6S$ [M + Na]$^+$: 568.1764; Found: 568.1737.

2-[(3-Bromobenzo[*b*]thiophen-2-yl)methyl]-1,3-dioxolane (16)

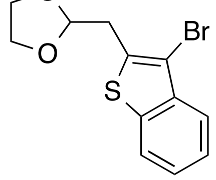

To a THF (6.0 mL) solution of $Ph_3P^+CH_2OCH_3Cl^−$ (1.55 g, 4.52 mmol) was added NaO*t*-Bu (566 mg, 5.89 mmol) at 0 °C. After stirring at the same temperature for 30 min, a THF (2.0 mL) and dichloromethane (3.0 mL) solution of 3-bromobenzo[*b*]thiophene-2-carbaldehyde (718 mg, 2.98 mmol) was added. After stirring at the same temperature for another 2 h, the reaction was quenched with H_2O, and organic materials were extracted with ethyl acetate three times. The combined extracts were washed with brine and dried over Na_2SO_4. After removal of the solvent under reduced pressure, ethylene glycol (367 mg, 5.92 mmol) and TsOH·H_2O (565 mg, 2.97 mmol) were added to a toluene (50 mL) solution of the obtained crude mixture. After stirring at 150 °C for 3 h in a reaction vessel equipped with a Dean–Stark apparatus, aqueous $NaHCO_3$ was added to the reaction mixture. The organic layer was separated and the aqueous layer was extracted with ethyl acetate three times. The combined extracts were washed with brine and dried over Na_2SO_4. After removal of the solvent under reduced pressure, the residue was purified by silica gel column chromatography (hexane/EtOAc = 10:1) to give **16** (709 mg, 80%) as a yellow oil.

^{1}H NMR (500 MHz, $CDCl_3$): δ 3.36 (d, *J* = 4.4 Hz, 2H), 3.83–4.07 (m, 4H), 5.23 (t, *J* = 4.4 Hz, 1H), 7.34 (ddd, *J* = 7.8, 7.6, 1.2 Hz, 1H), 7.41 (ddd, *J* = 7.8, 7.5, 1.0 Hz, 1H), 7.75 (d, *J* = 7.6 Hz, 1H), 7.76 (d, *J* = 7.5 Hz, 1H). ^{13}C NMR (126 MHz, $CDCl_3$): δ 34.9, 65.2, 102.6, 108.0, 122.1, 122.9, 124.8, 125.0, 133.4, 137.8, 138.0. IR (neat): ν 3060, 2960, 2883, 1433, 1394, 1304, 1252, 1209, 1124, 1018, 1007, 984, 943, 920, 847, 748, 725, 714, 673, 604, 517 cm^{-1}. HRMS (EI) *m*/*z* Calcd. for $C_{12}H_{11}{}^{79}BrO_2S$ $[M]^+$: 297.9658; Found: 297.9656.

2-[(2'-Bromo-[1,1'-biphenyl]-2-yl)methyl]-1,3-dioxolane

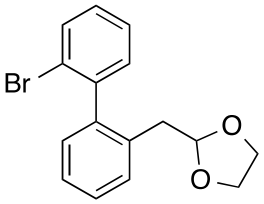

A dioxane (31.7 mL) and H_2O (15.8 mL) solution of 1-bromo-2-iodobenzene (2.69 g, 9.50 mmol), boronate ester **3** (2.90 g, 9.99 mmol), $Pd_2(dba)_3 \cdot CHCl_3$ (160 mg, 0.15 mmol), PPh_3 (162 mg, 0.62 mmol), and K_3PO_4 (8.04 g, 37.9 mmol) was degassed by using the freeze-pump-thaw method three times. After stirring at 120 °C for 2 h, ethyl acetate was added to the mixture, and organic materials were extracted with ethyl acetate three times. The combined extracts were washed with brine and dried over Na_2SO_4. After removal of the solvent under reduced pressure, the residue was purified by silica gel column chromatography (hexane/EtOAc = 10:1) to give 2-[(2'-bromo-[1,1'-biphenyl]-2-yl)methyl]-1,3-dioxolane (2.68 g, 88%) as a yellow oil.

^{1}H NMR (500 MHz, $CDCl_3$): δ 2.74 (dd, *J* = 14.3, 5.2 Hz, 1H), 2.85 (dd, *J* = 14.3, 5.0 Hz, 1H), 3.70–3.92 (m, 4H), 4.93 (dd, *J* = 5.2, 5.0 Hz, 1H), 7.13 (d, *J* = 7.5 Hz, 1H), 7.17–7.40 (m, 5H), 7.46 (d, *J* = 7.6 Hz, 1H), 7.64 (d, *J* = 8.0 Hz, 1H). ^{13}C NMR (126 MHz, $CDCl_3$): δ 37.7, 64.66, 64.73, 104.0, 123.8, 126.4, 127.0, 127.9, 128.8, 129.7, 130.2, 131.4, 132.5, 134.1, 141.4, 142.0. IR (neat): ν 3060, 2962, 2885, 1466, 1444, 1423, 1398, 1134, 1047, 1027, 1005, 985, 943, 754, 732, 660, 523 cm^{-1}. HRMS (EI) *m*/*z* Calcd. for $C_{16}H_{15}{}^{79}BrO_2$ $[M]^+$: 318.0250; Found: 318.0247.

2-{2'-[(1,3-Dioxolan-2-yl)methyl]-[1,1'-biphenyl]-2-yl}-4,4,5,5-tetramethyl-1,3,2-dioxaborolane (17)

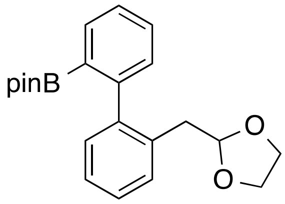

A 1,4-dioxane (42.0 mL) solution of 2-[(2'-bromo-[1,1'-biphenyl]-2-yl)methyl]-1,3-dioxolane (4.33 g, 13.6 mmol), B_2pin_2 (3.90 g, 15.4 mmol), $PdCl_2(dppf)\cdot CH_2Cl_2$ (115 mg, 0.140 mmol) and KOAc (5.50 g, 56.0 mmol) was degassed by using the freeze-pump-thaw method three times. After stirring at 120 °C for 14 h, ethyl acetate and water were added to the mixture, and organic materials were extracted with ethyl acetate three times. The combined extracts were washed with brine and dried over Na_2SO_4. After removal of the solvent under reduced pressure, the residue was purified by silica gel column chromatography (toluene/EtOAc = 50:1) to give **17** (2.55 g, 51%) as a yellow oil.

^{1}H NMR (500 MHz, $CDCl_3$): δ 1.04 (s, 6H), 1.07 (s, 6H), 2.82 (d, *J* = 5.2 Hz, 2H), 3.68–3.89 (m, 4H), 4.90 (t, *J* = 5.2 Hz, 1H), 7.11 (d, *J* = 7.5 Hz, 1H), 7.18 (dd, *J* = 7.4, 7.4 Hz, 1H), 7.22 (d, *J* = 7.6 Hz, 1H), 7.27 (dd, *J* = 7.6, 7.4 Hz, 1H), 7.31 (dd, *J* = 7.5, 7.4 Hz, 1H), 7.36 (d, *J* = 7.6 Hz, 1H), 7.39–7.42 (m, 1H), 7.72 (d, *J* = 7.4 Hz, 1H). ^{13}C NMR (126 MHz, $CDCl_3$): δ 24.3, 24.4, 37.8, 64.5, 64.57, 64.62, 83.2, 104.3, 125.4, 126.1, 126.8, 129.3, 129.5, 129.7, 129.9, 134.0, 134.1, 143.6, 147.0. IR (neat): ν 3060, 2981, 2931, 2883, 1595, 1479, 1437, 1381, 1352, 1315, 1269, 1144, 1119, 1078, 1039, 860, 754, 667 cm^{-1}. HRMS (EI) *m*/*z* Calcd. for $C_{22}H_{27}BO_4$ [M]$^+$: 366.1997; Found: 366.2015.

2-[(3-{2'-[(1,3-Dioxolan-2-yl)methyl]-[1,1'-biphenyl]-2-yl}benzo[*b*]thiophen-2-yl)methyl]-1,3-dioxolane (18)

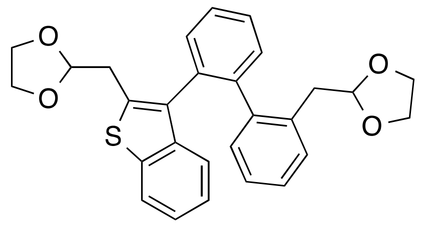

A dioxane (1.6 mL) and H_2O (0.8 mL) solution of 2-[(3-bromobenzo[*b*]thiophen-2-yl)methyl]-1,3-dioxolane (**16**, 140 mg, 0.47 mmol), boronate ester **17** (190 mg, 0.52 mmol), $Pd_2(dba)_3·CHCl_3$ (12 mg, 12 μmol), SPhos (20 mg, 48 μmol), and K_3PO_4 (601 mg, 2.83 mmol) was degassed by using the freeze-pump-thaw method three times. After stirring at 120 °C for 9 h, ethyl acetate and water were added to the mixture, and organic materials were extracted with ethyl acetate three times. The combined extracts were washed with brine and dried over Na_2SO_4. After removal of the solvent under reduced pressure, the residue was purified by silica gel column chromatography (hexane/EtOAc = 3:1) to give **18** (199 mg, 92%) as a yellow oil.

^{1}H NMR (500 MHz, $CDCl_3$): δ 2.43 (dd, *J* = 14.4, 5.0 Hz, 0.5H), 2.63 (dd, *J* = 15.0, 3.9 Hz, 0.5H), 2.83–2.91 (m, 2H), 3.00 (dd, *J* = 14.4, 5.1 Hz, 0.5H), 3.05 (dd, *J* = 15.0, 4.7 Hz, 0.5H), 3.68–3.99 (m, 8H), 4.61 (dd, *J* = 3.9, 3.9 Hz, 0.5H), 4.80 (dd, *J* = 5.0, 5.0 Hz, 0.5H), 4.82 (dd, *J* = 4.6, 4.5 Hz, 0.5H), 5.10 (dd, *J* = 4.9, 4.9 Hz, 0.5H), 6.74–6.81 (m, 1H), 7.00–7.08 (m, 1.5H), 7.09–7.12 (m, 0.5H), 7.13–7.18 (m, 1.5H), 7.20–7.27 (m, 1.5H), 7.33–7.37 (m, 1H), 7.39–7.49 (m, 3.5H), 7.52–7.55 (m, 0.5H), 7.62–7.65 (m, 0.5H), 7.67–7.71 (m, 0.5H). ^{13}C NMR (126 MHz, $CDCl_3$): δ 34.0, 34.5, 37.1, 37.3, 64.5, 64.6, 64.66, 64.75, 64.8, 65.01, 65.05, 103.3, 103.4, 104.5, 104.8, 121.6, 121.7, 122.7, 123.0, 123.5, 123.6, 123.7, 125.56, 125.61, 126.91, 126.95, 127.2, 127.3, 127.4, 127.7, 129.4, 129.7, 130.3, 131.5, 131.6, 131.7, 132.0, 133.5, 133.9, 134.2, 134.3, 134.57, 134.58, 134.7, 135.0, 138.8, 139.5, 140.57, 140.63, 141.0, 141.4, 141.6. IR

(neat): ν 3055, 2952, 2884, 1473, 1456, 1435, 1396, 1039, 1007, 984, 943, 752, 735 cm^{-1}. HRMS (APCI+) *m*/*z* Calcd. for $C_{28}H_{27}O_4S$ [M + H]$^+$: 459.1625; Found: 459.1648.

3. Synthesis of helicenes

Benzo[*a*]naphtho[1,2-*k*]tetraphene (5a)

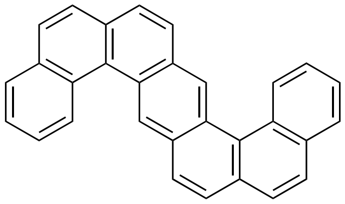

Compound **5a** was synthesized by the method described for compound **1a** using bisacetal **8a** (70 mg, 0.14 mmol), trifluoromethanesulfonic acid (3.2 mg, 21 μmol), and HFIP (0.5 mL) at 0 ºC for 40 min. Purification by PTLC (hexane/CH_2Cl_2 = 1:1) gave **5a** (48 mg, 90%) as a yellow solid.

^{1}H NMR (500 MHz, $CDCl_3$): δ 7.66 (dd, *J* = 7.4, 7.2 Hz, 2H), 7.76 (dd, *J* = 8.4, 7.2 Hz, 2H), 7.79 (d, *J* = 8.6 Hz, 2H), 7.85 (d, *J* = 8.4 Hz, 2H), 7.96 (d, *J* = 8.4 Hz, 2H), 8.02 (d, *J* = 8.6 Hz, 2H), 8.06 (d, *J* = 7.4 Hz, 2H), 9.31 (d, *J* = 8.4 Hz, 2H), 9.58 (s, 2H). ^{13}C NMR (126 MHz, $CDCl_3$): δ 125.8, 126.6, 126.8, 127.1, 127.2, 127.5, 127.7, 127.9, 128.2, 128.6, 128.7, 130.4, 131.0, 131.7, 133.5.

Spectral data for this compound showed good agreement with literature data [11].

Benzo[*a*]naphtho[2,1-*m*]tetraphene (5b)

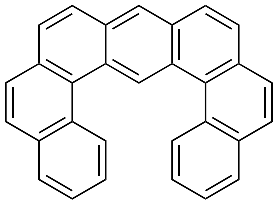

Compound **5b** was synthesized by the method described for compound **1a** using bisacetal **8b** (101 mg, 0.20 mmol), trifluoromethanesulfonic acid (4.7 mg, 31 μmol), and HFIP (0.7 mL) at 0 °C for 40 min. Purification by silica gel column chromatography (hexane/CH_2Cl_2 = 3:1) gave **5b** (65 mg, 86%) as a yellow solid.

^{1}H NMR (500 MHz, $CDCl_3$): δ 7.56–7.67 (m, 4H), 7.80 (d, *J* = 8.6 Hz, 2H), 7.83 (d, *J* = 8.4 Hz, 2H), 7.93 (d, *J* = 8.3 Hz, 2H), 7.98–8.06 (m, 4H), 8.55 (s, 1H), 9.21 (d, *J* = 8.2 Hz, 2H), 10.96 (s, 1H). ^{13}C NMR (126 MHz, $CDCl_3$): δ 125.9, 126.5, 126.7, 127.0, 127.3, 127.4, 127.5, 127.76, 127.79, 127.82, 128.7, 128.9, 130.4, 131.3, 131.6, 133.8. IR (neat): ν 3047, 1153, 924, 881, 825, 739 cm^{-1}. HRMS (APCI+): *m*/*z* Calcd. for $C_{30}H_{19}$ $[M + H]^+$: 379.1481; Found: 379.1497.

Spectral data for this compound showed good agreement with literature data [12].

Naphtho[2,1-*b*]benzofuran (9a)

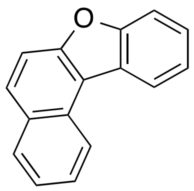

Compound **9a** was synthesized by the method described for compound **1a** using acetal **11a** (46 mg, 0.16 mmol), trifluoromethanesulfonic acid (2.4 mg, 16 μmol), and HFIP (0.5 mL) at 0 °C for 40 min. Purification by silica gel column chromatography (hexane/CH_2Cl_2 = 3:1) gave **9a** (34 mg, 96%) as a pale yellow solid.

^{1}H NMR (500 MHz, $CDCl_3$): δ 7.43–7.54 (m, 3H), 7.67–7.71 (m, 2H), 7.74 (d, *J* = 8.9 Hz, 1H), 7.89 (d, *J* = 8.9 Hz, 1H), 7.99 (d, *J* = 8.2 Hz, 1H), 8.36 (d, *J* = 7.2 Hz, 1H), 8.59 (d, *J* = 8.3 Hz, 1H). ^{13}C NMR (126 MHz, $CDCl_3$): δ 111.9, 112.7, 117.3, 121.9, 123.1, 123.4, 124.4, 124.9, 125.9, 127.1, 128.5, 129.1, 129.2, 130.4, 154.3, 155.9.

Spectral data for this compound showed good agreement with literature data [13].

Benzo[*b*]naphtho[1,2-*d*]thiophene (9b)

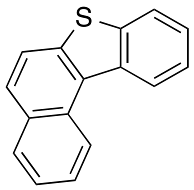

Compound **9b** was synthesized by the method described for compound **1a** using acetal **11b** (88 mg, 0.30 mmol), trifluoromethanesulfonic acid (4.9 mg, 33 μmol), and HFIP (1.0 mL) at 0 °C for 40 min. Purification by silica gel column chromatography (hexane/CH_2Cl_2 = 3:1) gave **9b** (61 mg, 88%) as a pale yellow solid.

^{1}H NMR (500 MHz, $CDCl_3$): δ 7.45 (dd, *J* = 8.1, 7.0 Hz, 1H), 7.51–7.56 (m, 2H), 7.74 (ddd, *J* = 8.4, 7.0, 1.2 Hz, 1H), 7.81 (d, *J* = 8.6 Hz, 1H), 7.85 (d, *J* = 8.6 Hz, 1H), 7.94–7.97 (m, 2H), 8.80 (d, *J* = 8.3 Hz, 1H), 8.95 (d, *J* = 8.5 Hz, 1H). ^{13}C NMR (126 MHz, $CDCl_3$): δ 121.0, 123.12, 123.14, 124.66, 124.74, 124.8, 125.1, 127.1, 127.8, 129.0, 129.4, 130.6, 131.9, 136.7, 138.6, 139.7.

Spectral data for this compound showed good agreement with literature data [14].

7-(4-Methylbenzenesulfonyl)-7*H*-benzo[*c*]carbazole (9c)

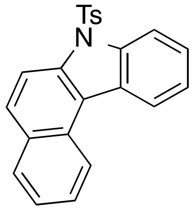

Compound **9c** was synthesized by the method described for compound **1a** using acetal **11c** (87 mg, 0.20 mmol), trifluoromethanesulfonic acid (3.0 mg, 20 μmol), and HFIP (0.7 mL) at 0 °C for 40 min. Purification by silica gel column chromatography (hexane/EtOAc = 10:1) gave **9c** (69 mg, 93%) as a pale yellow solid.

^{1}H NMR (500 MHz, $CDCl_3$): δ 2.18 (s, 3H), 7.02 (d, *J* = 8.5 Hz, 2H), 7.48–7.53 (m, 3H), 7.64–7.68 (m, 3H), 7.93 (d, *J* = 9.2 Hz, 1H), 7.98 (d, *J* = 8.2 Hz, 1H), 8.44 (d, *J* = 7.7

Hz, 1H), 8.51 (d, J = 8.4 Hz, 1H), 8.59 (d, J = 9.2 Hz, 1H), 8.68 (d, J = 8.4 Hz, 1H). ^{13}C NMR (126 MHz, $CDCl_3$): δ 21.3, 114.8, 115.3, 119.8, 122.1, 123.4, 124.2, 124.8, 126.1, 126.3, 127.0, 127.2, 128.5, 128.7, 129.0, 129.6, 130.9, 134.9, 136.5, 138.1, 144.9.

Spectral data for this compound showed good agreement with literature data. [15]

Dinaphtho[2,1-*b*:1',2'-*d*]furan (12a)

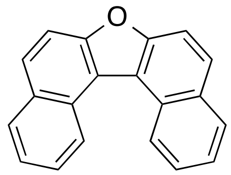

Compound **12a** was synthesized by the method described for compound **1a** using bisacetal **14a** (55 mg, 0.14 mmol), trifluoromethanesulfonic acid (3.7 mg, 25 μmol), and HFIP (0.5 mL) at room temperature for 40 min. Purification by silica gel column chromatography (hexane/CH_2Cl_2 = 2:1) gave **12a** (34 mg, 90%) as a brown solid.

^{1}H NMR (500 MHz, $CDCl_3$): δ 7.56 (ddd, J = 8.0, 6.9, 1.0 Hz, 1H), 7.72 (ddd, J = 8.5, 6.9, 1.4 Hz, 1H), 7.80 (d, J = 8.8 Hz, 1H), 7.92 (d, J = 8.8 Hz, 1H), 8.04 (d, J = 8.0 Hz, 1H), 9.13 (d, J = 8.5 Hz, 1H). ^{13}C NMR (126 MHz, $CDCl_3$): δ 112.7, 119.4, 124.4, 125.6, 126.1, 128.3, 128.6, 129.5, 131.2, 154.3.

Spectral data for this compound showed good agreement with literature data [16].

Dinaphtho[2,1-*b*:1',2'-*d*]thiophene (12b)

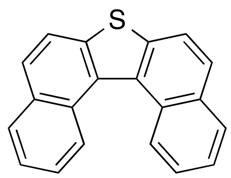

Compound **12b** was synthesized by the method described for compound **1a** using bisacetal **14b** (116 mg, 0.28 mmol), trifluoromethanesulfonic acid (4.5 mg, 30 μmol),

and HFIP (1.0 mL) at 0 °C for 40 min. Purification by silica gel column chromatography (hexane/EtOAc = 5:1) gave **12b** (73 mg, 90%) as a brown solid.

^{1}H NMR (500 MHz, $CDCl_3$): δ 7.56–7.58 (m, 4H), 7.92 (d, *J* = 8.6 Hz, 2H), 7.95 (d, *J* = 8.6 Hz, 2H), 8.02–8.04 (m, 2H), 8.86–8.88 (m, 2H). ^{13}C NMR (126 MHz, $CDCl_3$): δ 120.8, 124.9, 125.2, 126.1, 127.4, 128.7, 129.9, 131.4, 132.1, 138.5.

Spectral data for this compound showed good agreement with literature data [17].

7-(4-Methylbenzenesulfonyl)-7*H*-dibenzo[*c*,*g*]carbazole (12c)

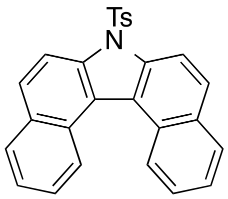

Compound **12c** was synthesized by the method described for compound **1a** using bisacetal **14c** (95 mg, 0.17 mmol), trifluoromethanesulfonic acid (3.8 mg, 25 μmol), and HFIP (0.6 mL) at room temperature for 40 min. Purification by silica gel column chromatography (hexane/CH_2Cl_2 = 2:1) gave **12c** (64 mg, 88%) as a yellow solid.

mp: 174.2–176.1 °C. ^{1}H NMR (500 MHz, $CDCl_3$): δ 2.13 (s, 3H), 7.00 (d, *J* = 8.4 Hz, 2H), 7.52 (dd, *J* = 8.1, 7.0 Hz, 2H), 7.56 (dd, *J* = 8.4, 7.0 Hz, 2H), 7.68 (d, *J* = 8.5 Hz, 2H), 7.94 (d, *J* = 9.1 Hz, 2H), 7.98 (d, *J* = 8.1 Hz, 2H), 8.65 (d, *J* = 9.1 Hz, 2H), 8.90 (d, *J* = 8.5 Hz, 2H). ^{13}C NMR (126 MHz, $CDCl_3$): δ 21.4, 114.6, 121.7, 124.9, 125.5, 125.8, 126.3, 127.9, 128.1, 128.7, 129.7, 131.4, 135.1, 136.3, 145.0. IR (neat): ν 3050, 2927, 2854, 1595, 1516, 1444, 1381, 1348, 1246, 1196, 1188, 1173, 1163, 1099, 820, 806, 768, 744, 678, 663, 615, 565, 540 cm^{-1}. HRMS (APCI+) *m*/*z* Calcd. for $C_{27}H_{20}NO_2S$ $[M + H]^+$: 422.1209; Found: 422.1206.

Benzo[*b*]benzo[5,6]phenanthro[4,3-*d*]thiophene (15)

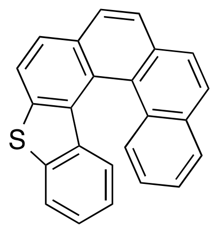

Compound **15** was synthesized by the method described for compound **1a** using bisacetal **18** (94 mg, 0.21 mmol), trifluoromethanesulfonic acid (6.2 mg, 41 μmol) and HFIP (0.9 mL) at room temperature for 40 min. Purification by silica gel column chromatography (hexane/CH_2Cl_2 = 3:1) to give **15** (43 mg, 63%) as a yellow solid.

mp: 175.4–177.0 °C. ^{1}H NMR (500 MHz, $CDCl_3$): δ 6.77 (dd, *J* = 8.2, 7.2 Hz, 1H), 6.91 (d, *J* = 8.3 Hz, 1H), 7.02 (dd, *J* = 8.2, 7.5 Hz, 1H), 7.23 (dd, *J* = 7.9, 7.2 Hz, 1H), 7.42 (dd, *J* = 7.7, 7.5 Hz, 1H), 7.85–7.87 (m, 2H), 7.90–8.00 (m, 6H), 8.05 (d, *J* = 8.4 Hz, 1H). ^{13}C NMR (126 MHz, $CDCl_3$): δ 121.5, 122.1, 122.4, 124.7, 125.3, 125.5, 125.6, 125.8, 125.9, 126.1, 126.3, 126.9, 127.4, 127.7, 128.1, 128.6, 130.2, 131.4, 131.51, 131.54, 132.1, 136.1, 138.4, 139.4. IR (neat): ν 3047, 1577, 1485, 1442, 1401, 1363, 1336, 1307, 1246, 1217, 1193, 1136, 1120, 1066, 837, 744, 609, 519 cm^{-1}. HRMS (APCI+) *m*/*z* Calcd. for $C_{24}H_{15}S$ $[M + H]^+$: 335.0889; Found: 335.0897.

4. References

1. Weimar, M.; Dürner, G.; Bats, J. W.; Göbel, M. W. *J. Org. Chem.*, **2010**, *75*, 2718–2721.
2. Takahashi, I.; Hayashi, M.; Fujita, T.; Ichikawa, J. *Chem. Lett.*, **2017**, *46*, 392–394.
3. Xie, Z.; Yang, B.; Liu, L.; Li, M.; Lin, D.; Ma, Y.; Cheng, G.; Liu, S. *J. Phys. Org. Chem.*, **2005**, *18*, 962–973.

4. Nakakuki, Y.; Hirose, T.; Matsuda, K. *J. Am. Chem. Soc.*, **2018**, *140*, 15461–15469.
5. Fredrich, S.; Bonasera, A.; Valderrey, V.; Hecht, S. *J. Am. Chem. Soc.*, **2018**, *140*, 6432–6440.
6. Dhiman, S.; Mishra, U. K.; Ramasastry, S. S. V. *Angew. Chem. Int. Ed.*, **2016**, *55*, 7737–7741.
7. Yao, C.-H.; Song, J.-S.; Chen, C.-T.; Yeh, T.-K.; Hsieh, T.-C.; Wu, S.-H.; Huang, C.-Y.; Huang, Y.-L.; Wang, M.-H.; Liu, Y.-W.; Tsai, C.-H.; Kumar, C. R.; Lee, J.-C. *Eur. J. Med. Chem.*, **2012**, *55*, 32–38.
8. Rennison, D.; Bova, S.; Cavalli, M.; Ricchelli, F.; Zulian, A.; Hopkins, B.; Brimble, M. A. *Bioorg. Med. Chem.*, **2007**, *15*, 2963–2974.
9. Nielsen, C. B.; Bjørnholm, T. *Org. Lett.*, **2004**, *6*, 3381–3384.
10. Zonta, C.; Fabris, F.; De Lucchi, O. *Org. Lett.*, **2005**, *7*, 1003–1006.
11. Talele, H. R.; Chaudhary, A. R.; Patel, P. R.; Bedekar, A. V. *Arkivoc*, **2011**, *9*, 15–37.
12. Mallory, F. B.; Mallory, C. W.; Sen Loeb, S. E. *Tetrahedron Lett.*, **1985**, *26*, 3773–3776.
13. Nervig, C. S.; Waller, P. J.; Kalyani, D. *Org. Lett.*, **2012**, *14*, 4838–4841.
14. Rafiq, S. M.; Sivasakthikumaran, R.; Mohanakrishnan, A. K. *Org. Lett.*, **2014**, *16*, 2720–2723.
15. Fan, X.; Yu, L.-Z.; Wei, Y.; Shi, M. *Org. Lett.*, **2017**, *19*, 4476–4479.
16. Ducos, P.; Liautard, V.; Robert, F.; Landais, Y. *Chem. Eur. J.*, **2015**, *21*, 11573–11578.
17. Sadorn, K.; Sinananwanich, W.; Areephong, J.; Nerungsi, C.; Wongma, C.; Pakawatchai, C.; Thongpanchang, T. *Tetrahedron Lett.*, **2008**, *49*, 4519–4521.

5. ^{1}H and ^{13}C NMR spectra

[5]Helicene (1a)

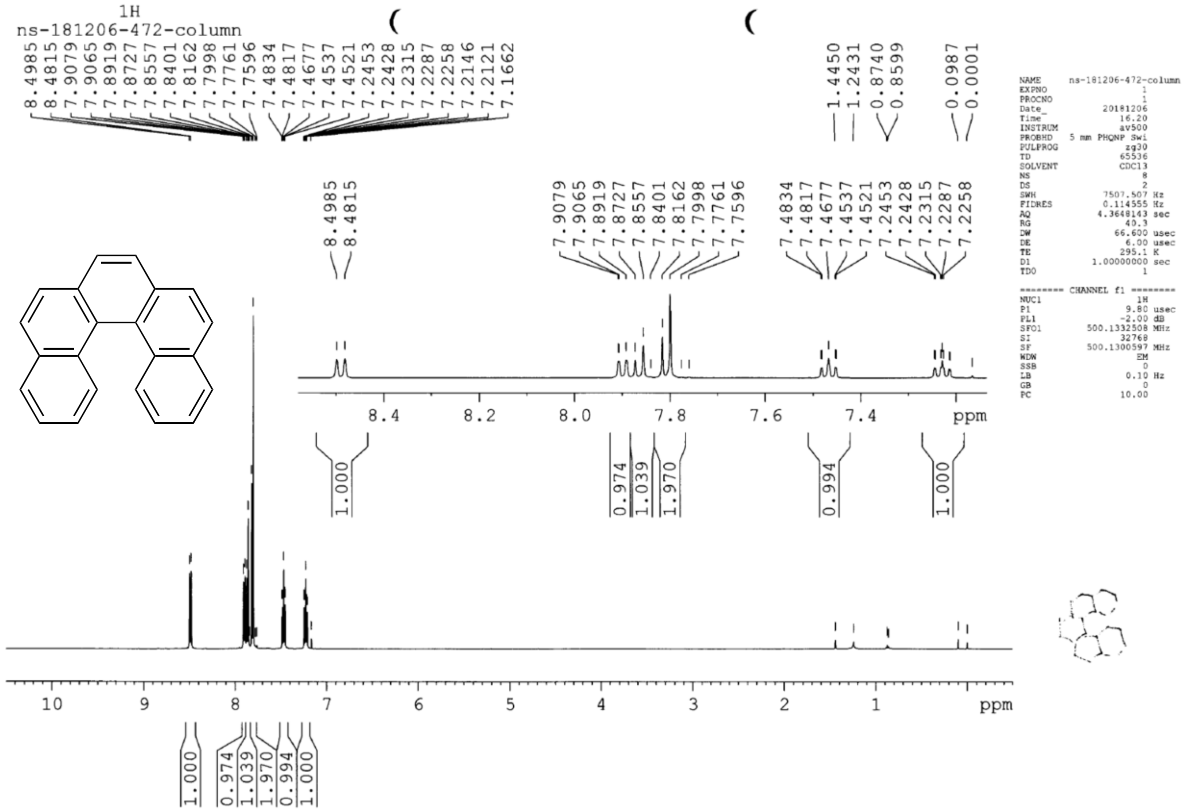

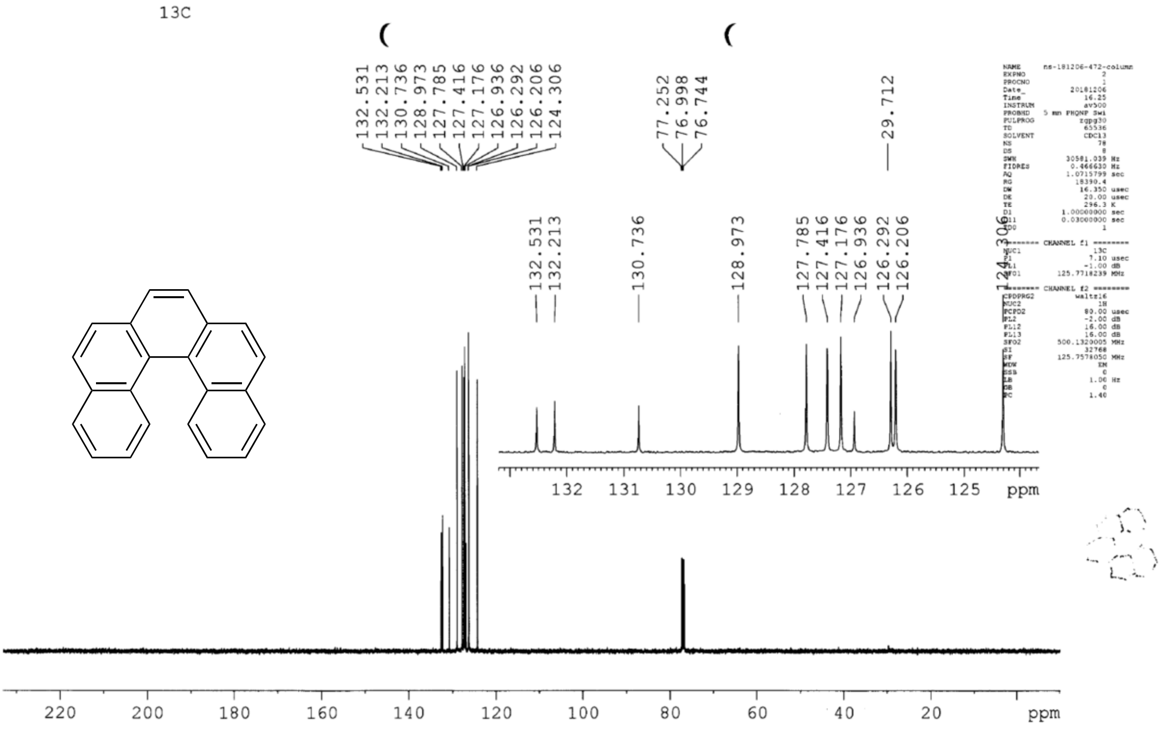

[6]Helicene (1b)

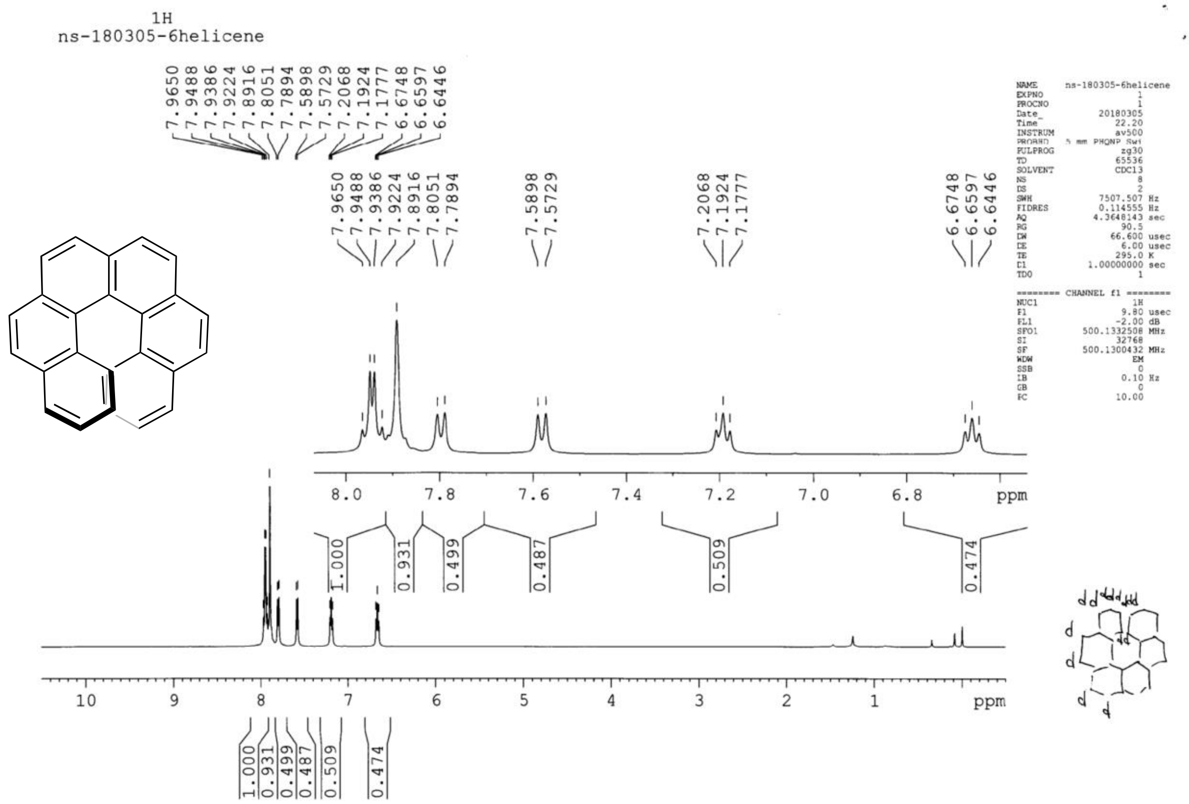

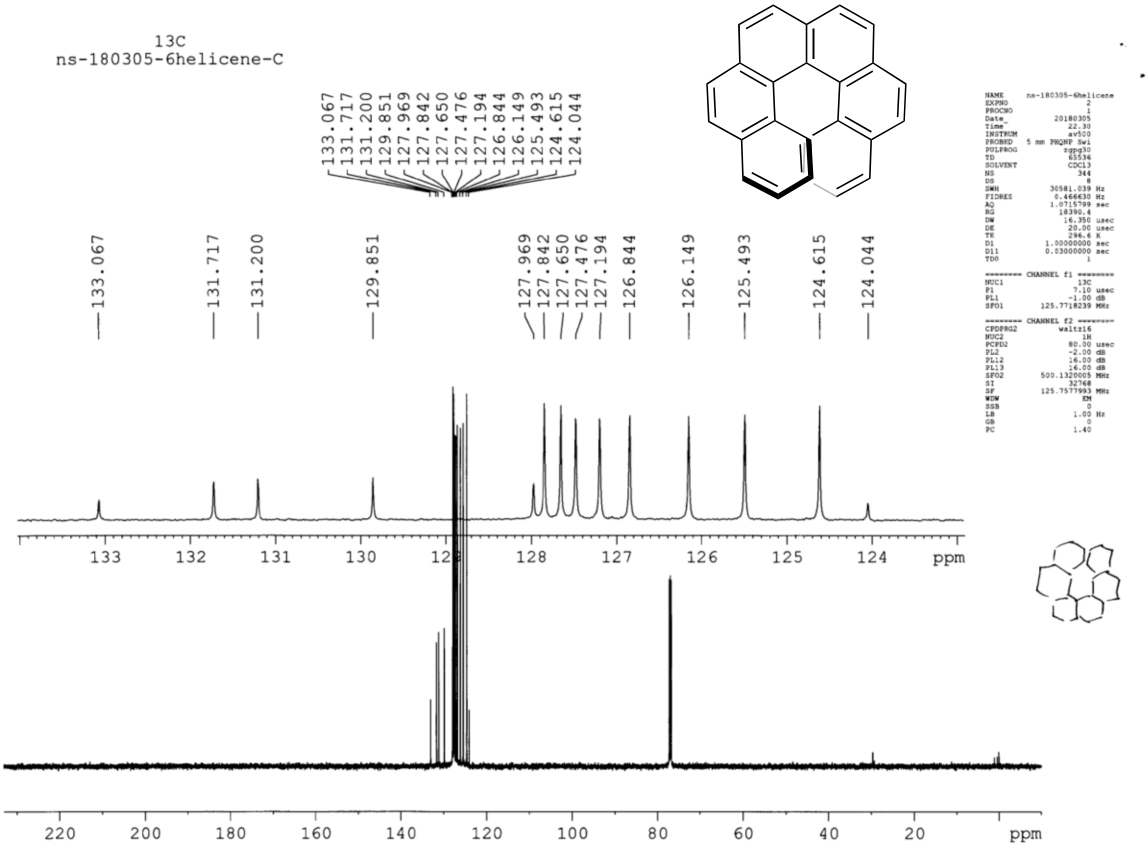

Benzo[*a*]naphtho[1,2-*k*]tetraphene (5a)

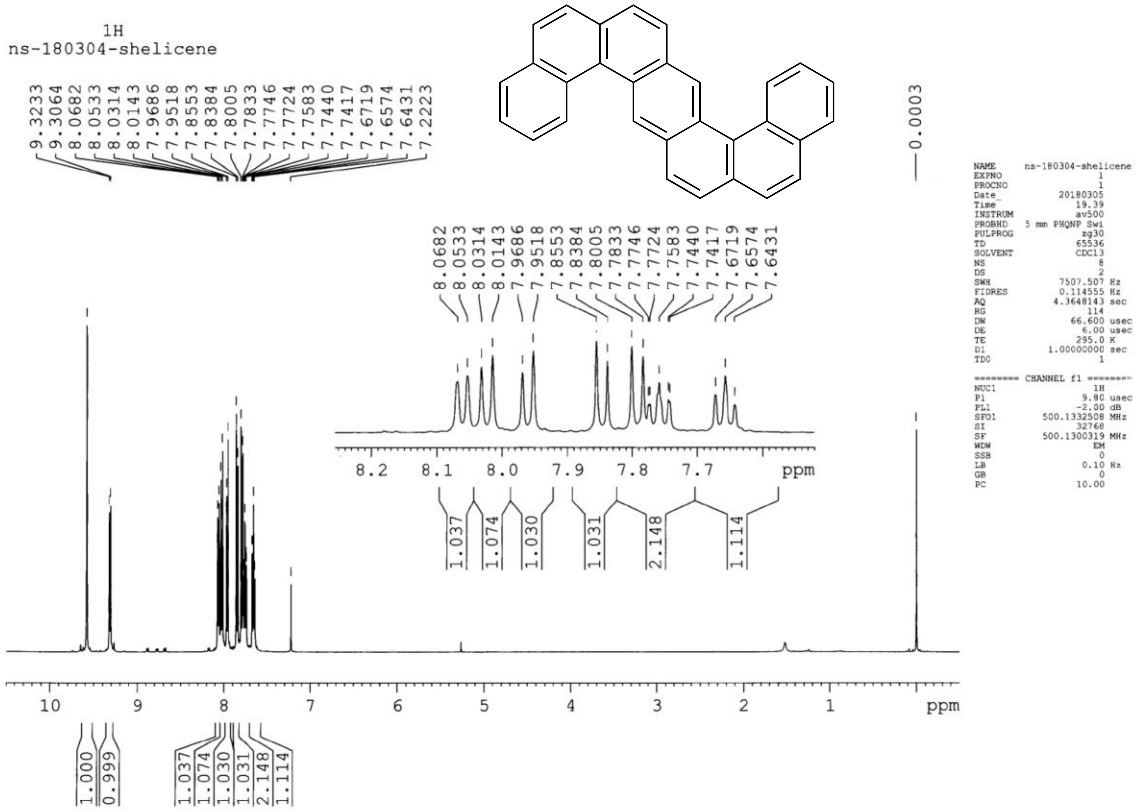

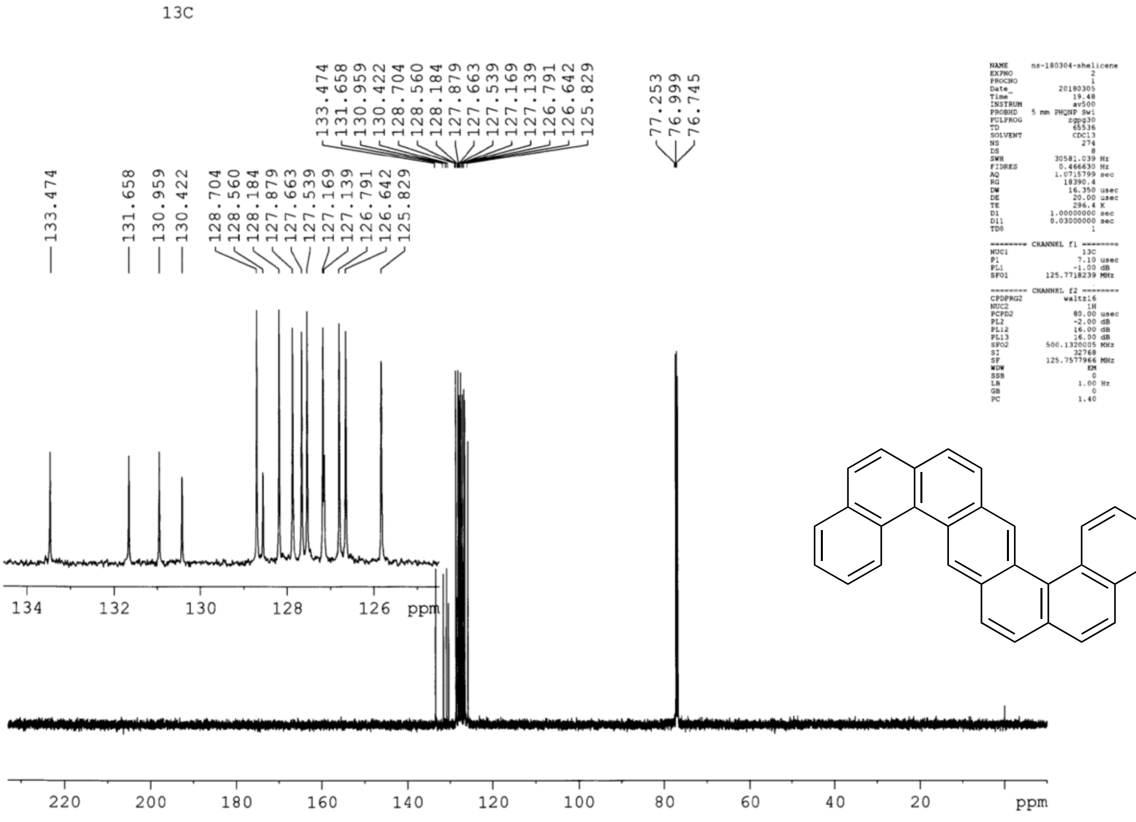

Benzo[*a*]naphtho[2,1-*m*]tetraphene (5b)

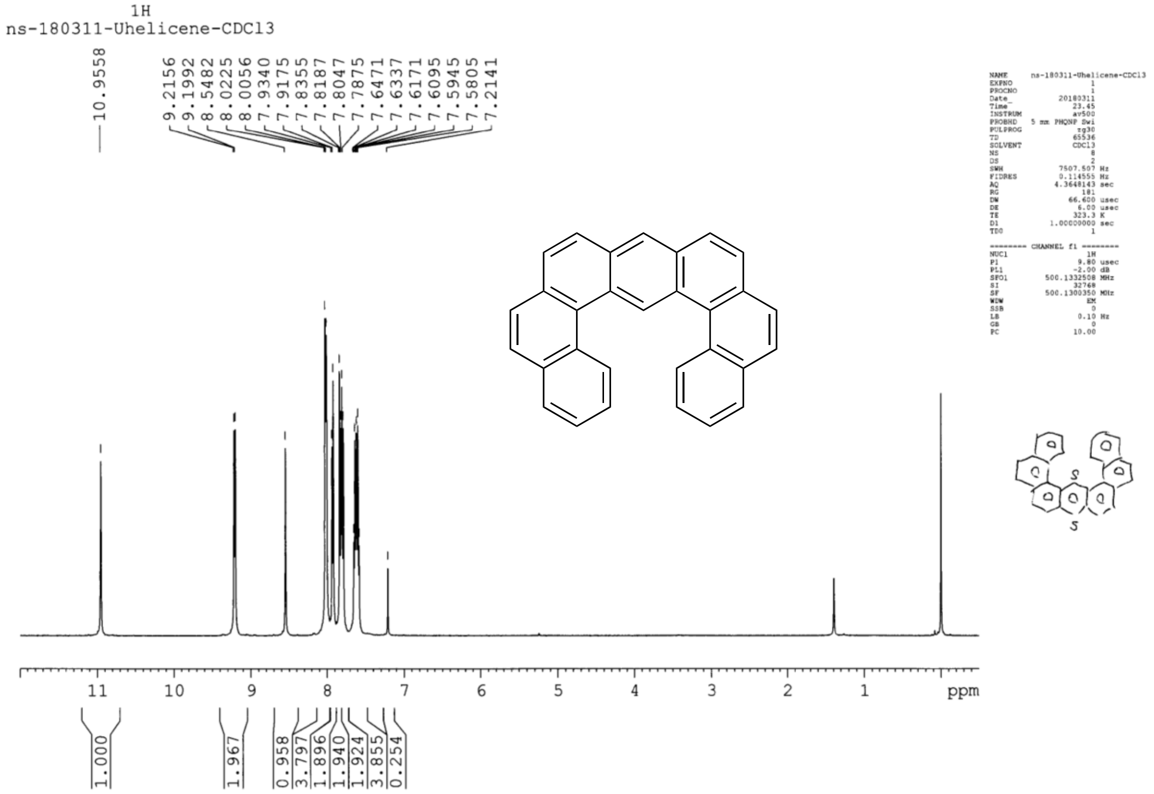

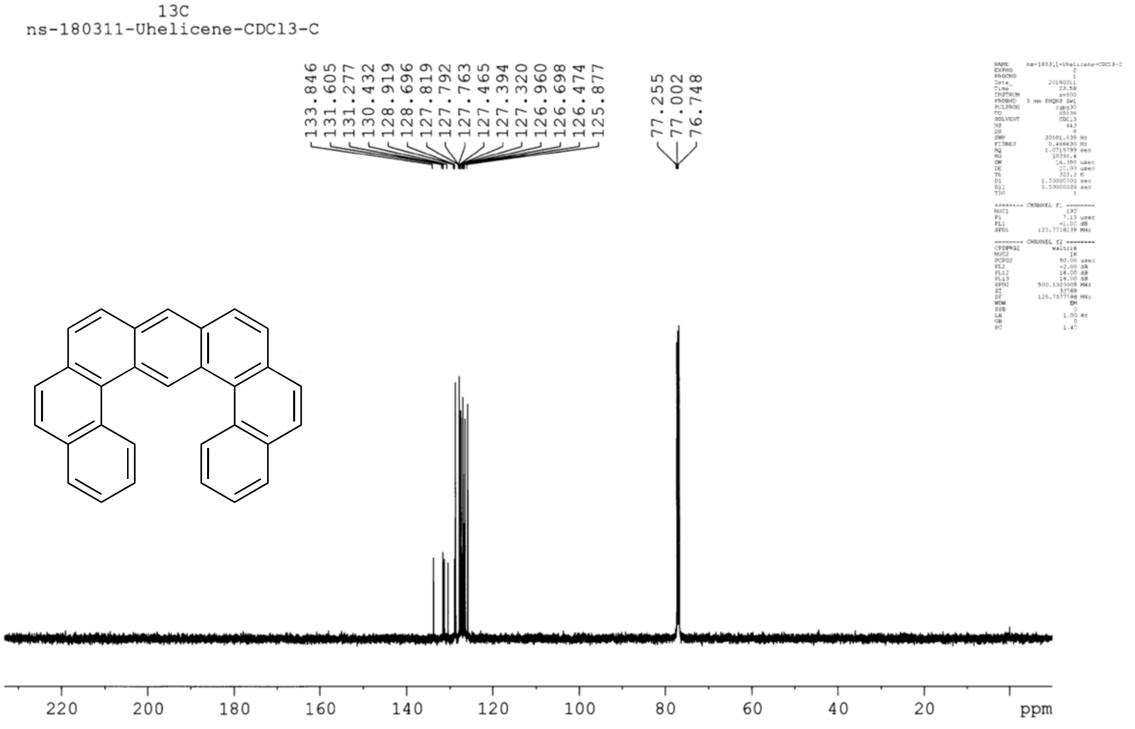

Naphtho[2,1-*b*]benzofuran (9a)

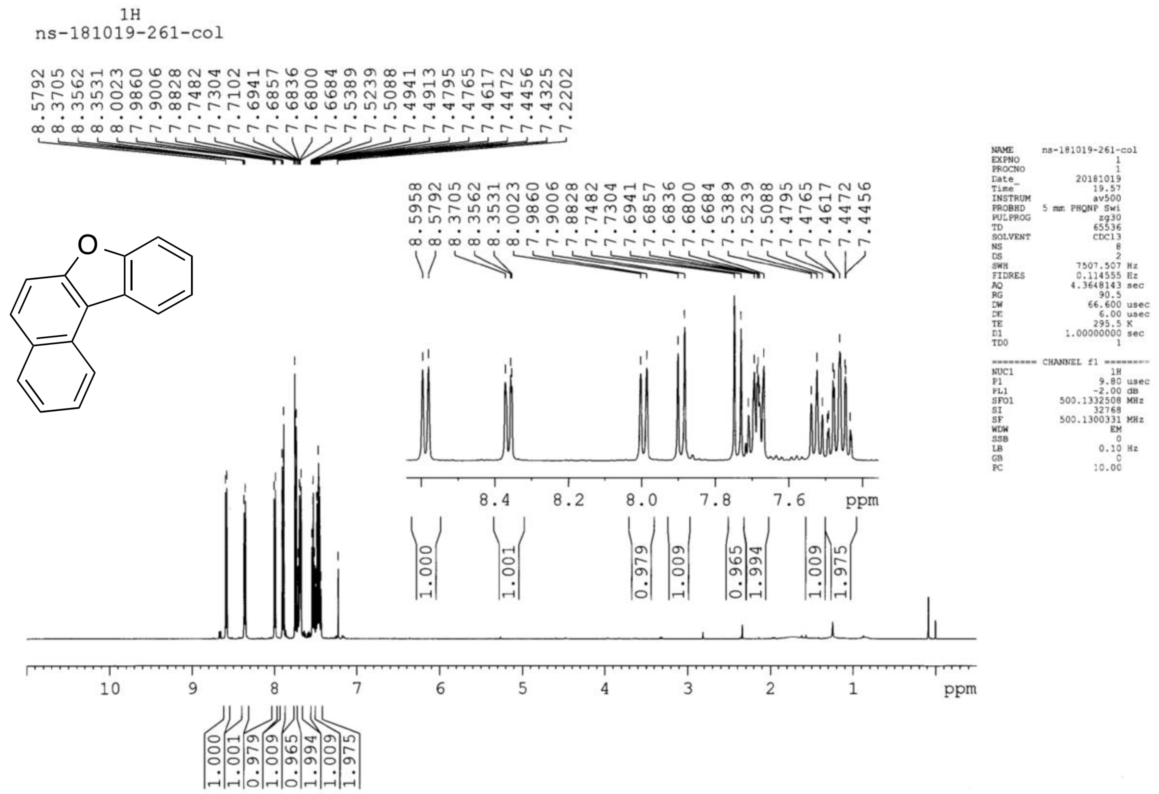

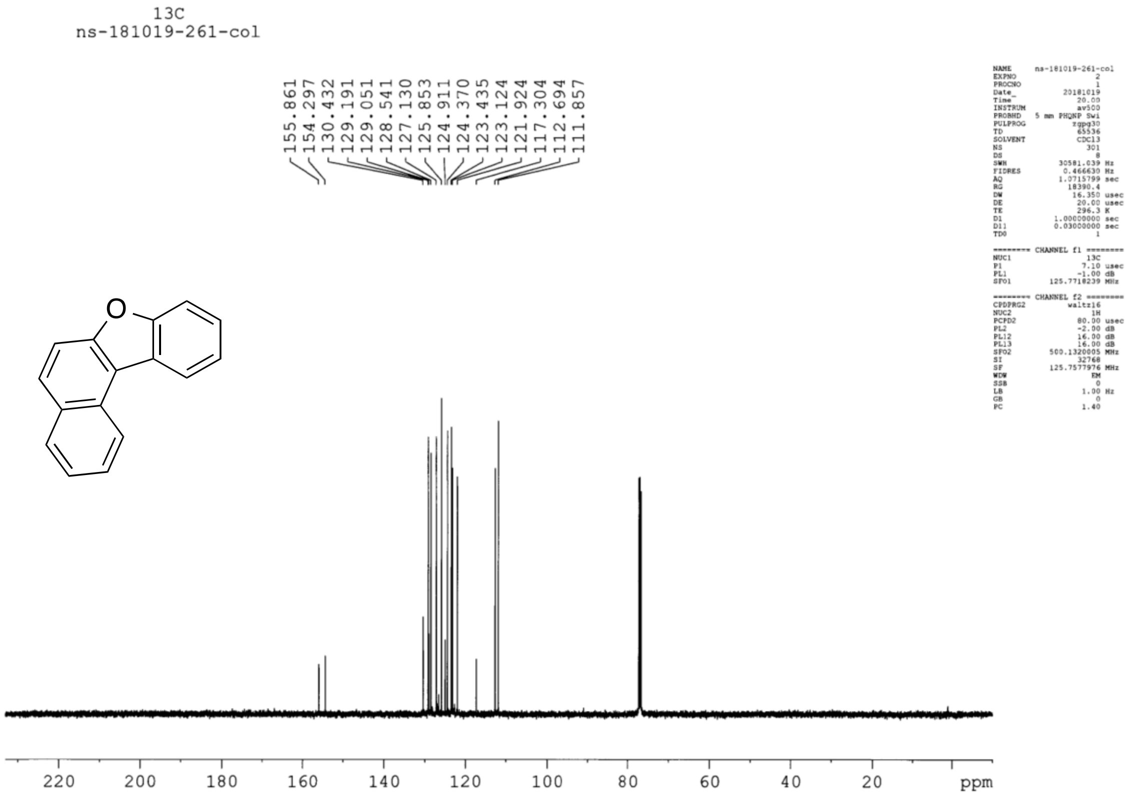

Benzo[*b*]naphtho[1,2-*d*]thiophene (9b)

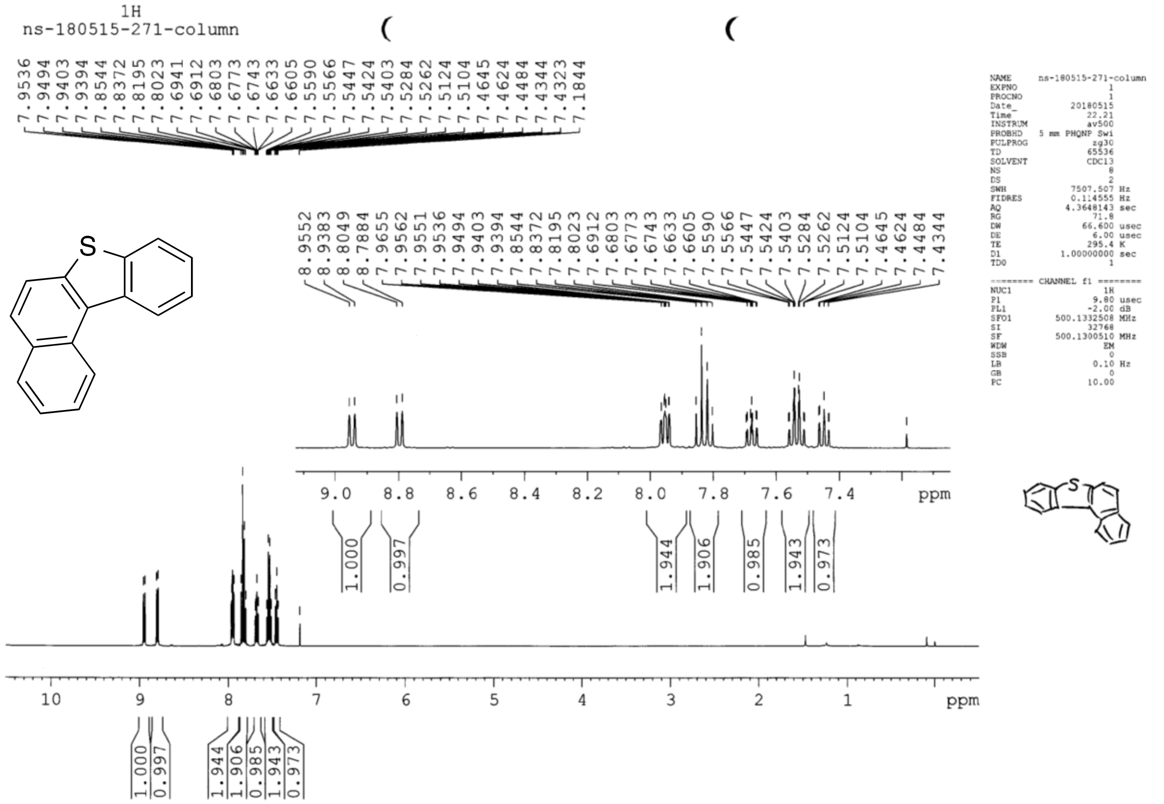

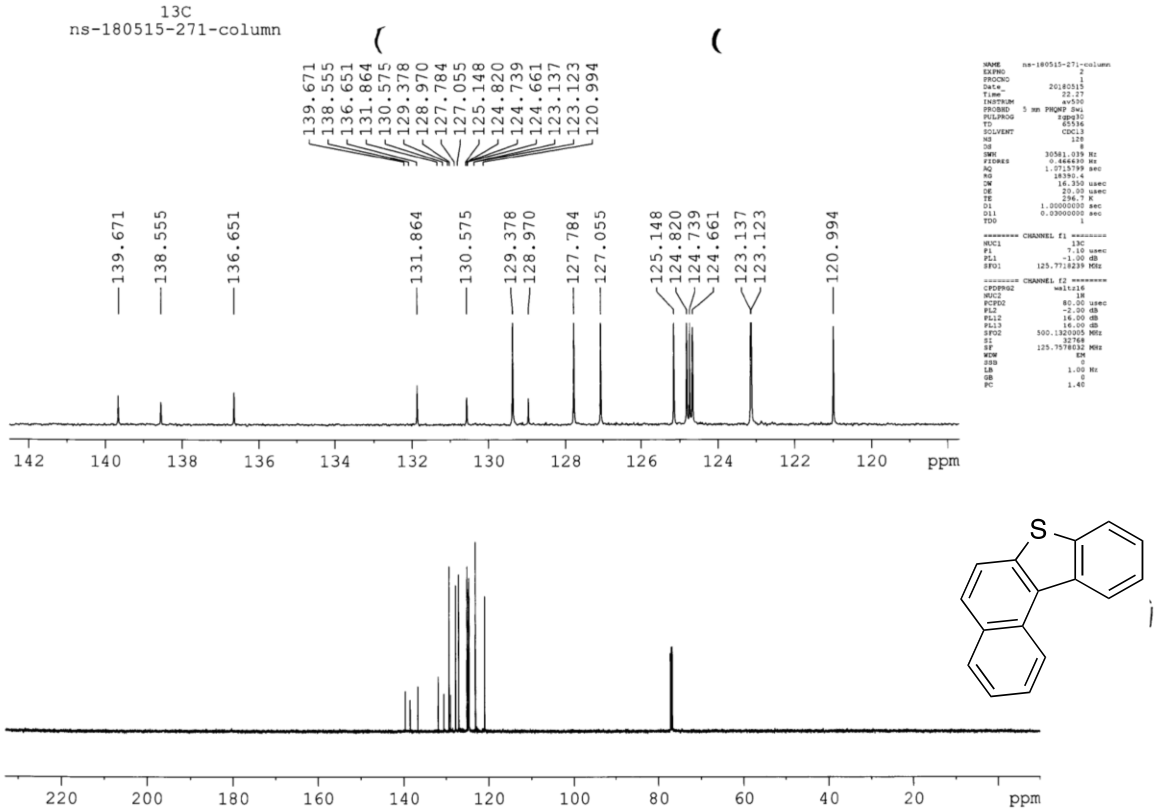

7-(4-Methylbenzenesulfonyl)-7*H*-benzo[*c*]carbazole (9c)

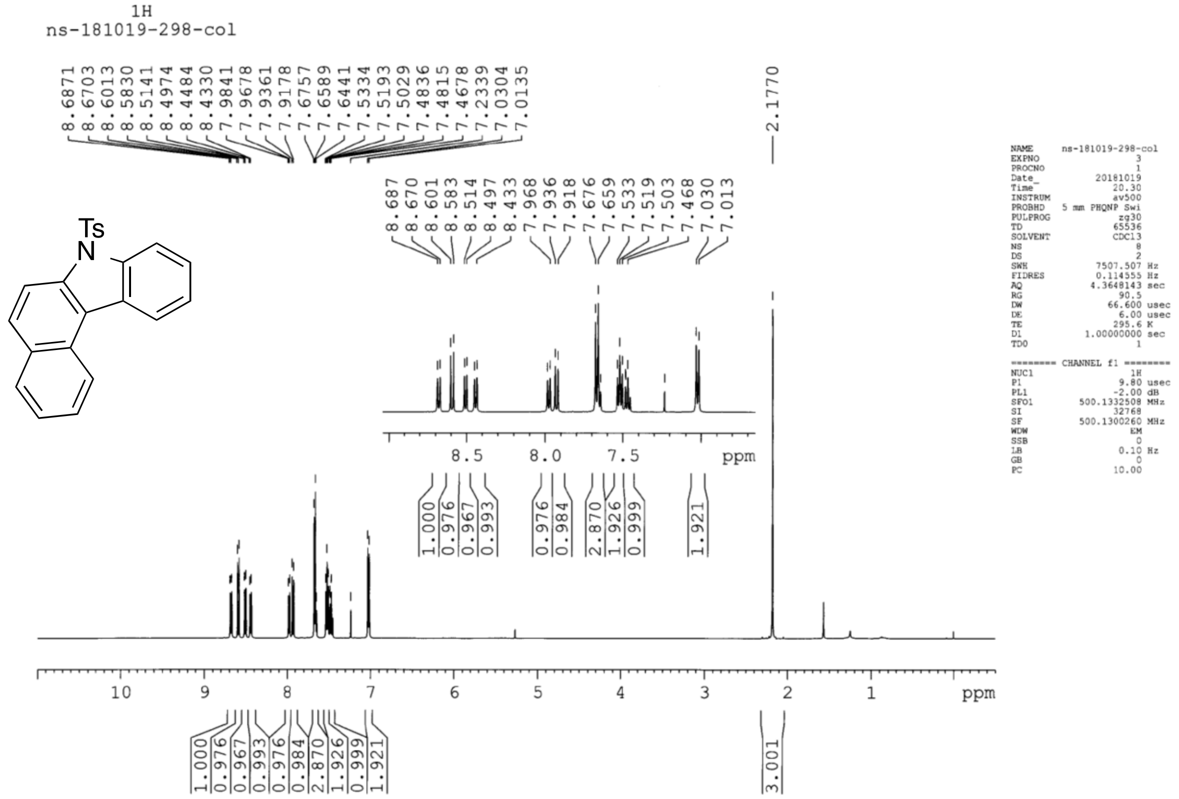

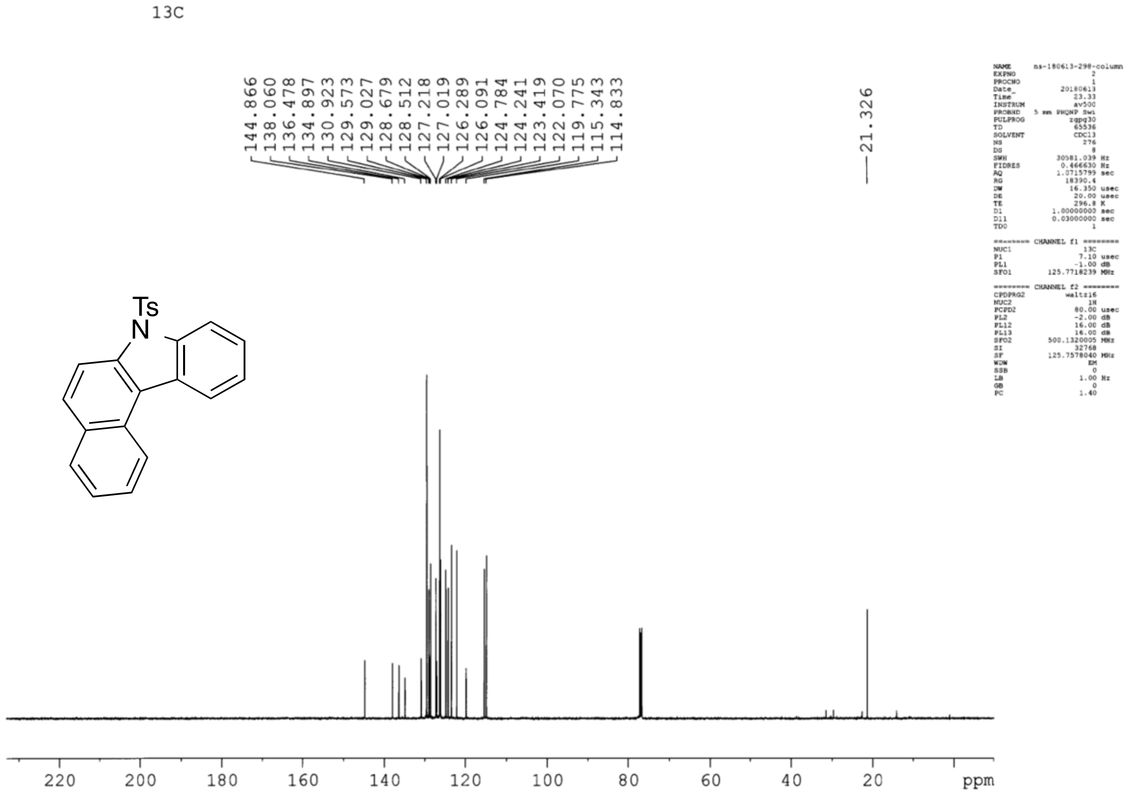

Dinaphtho[2,1-*b*:1',2'-*d*]furan (12a)

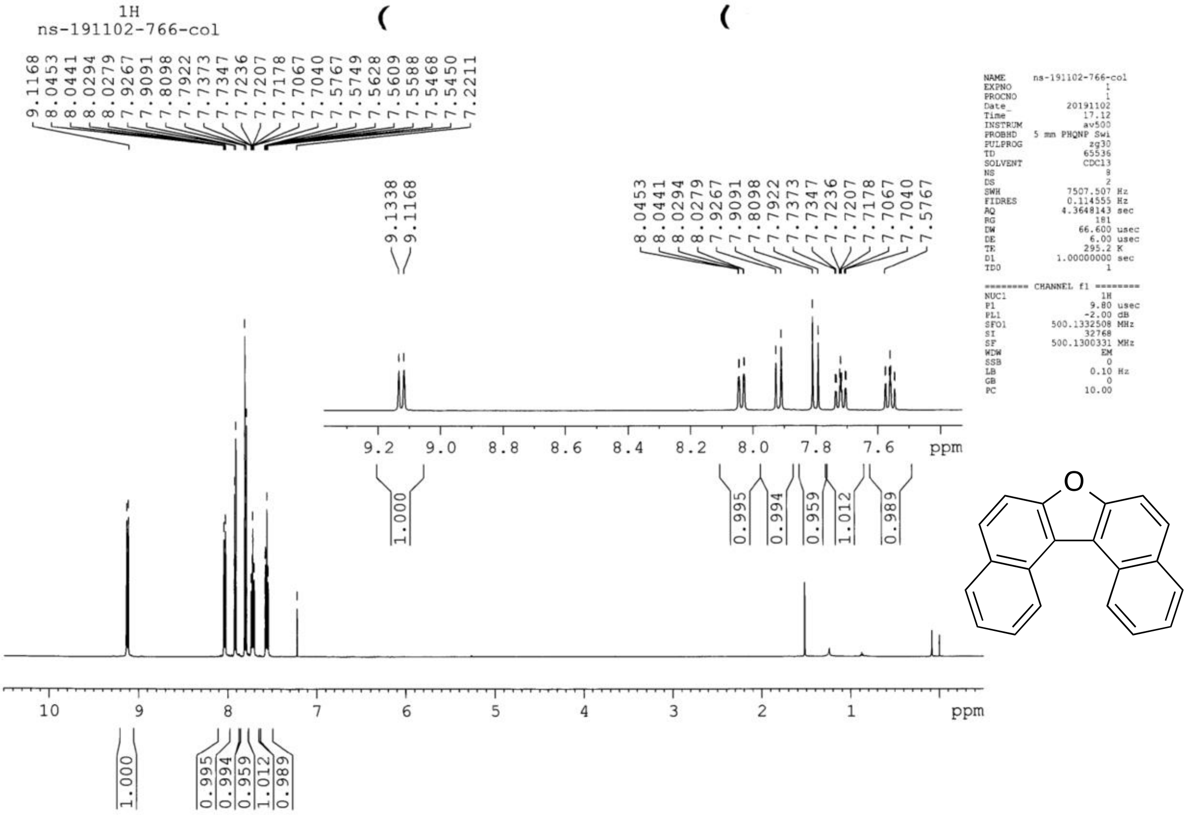

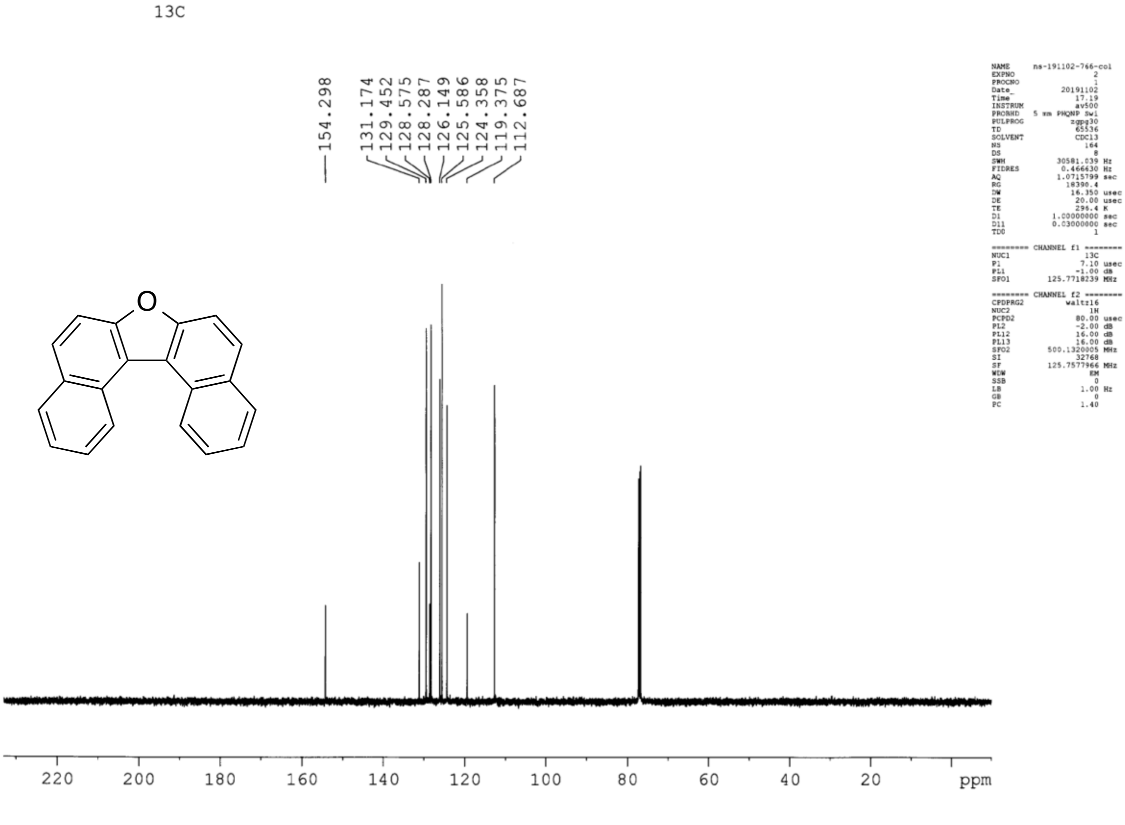

Dinaphtho[2,1-*b*:1',2'-*d*]thiophene (12b)

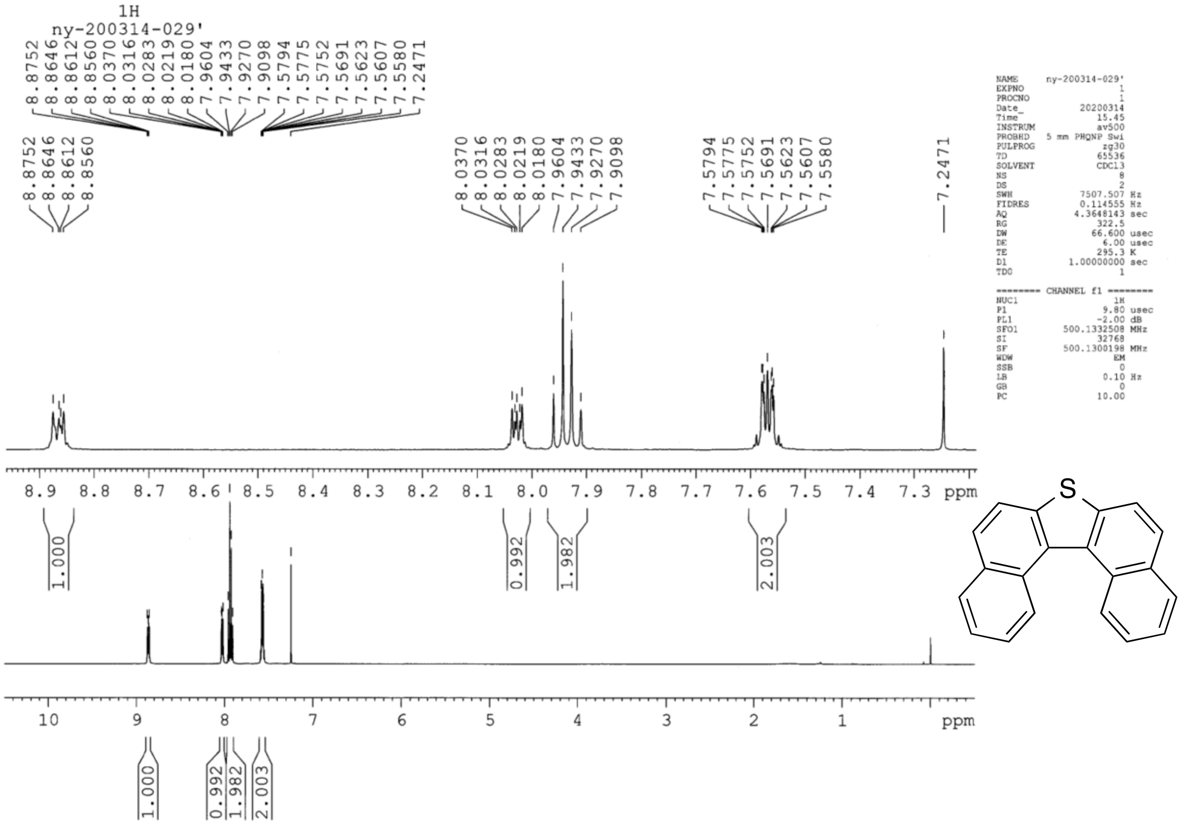

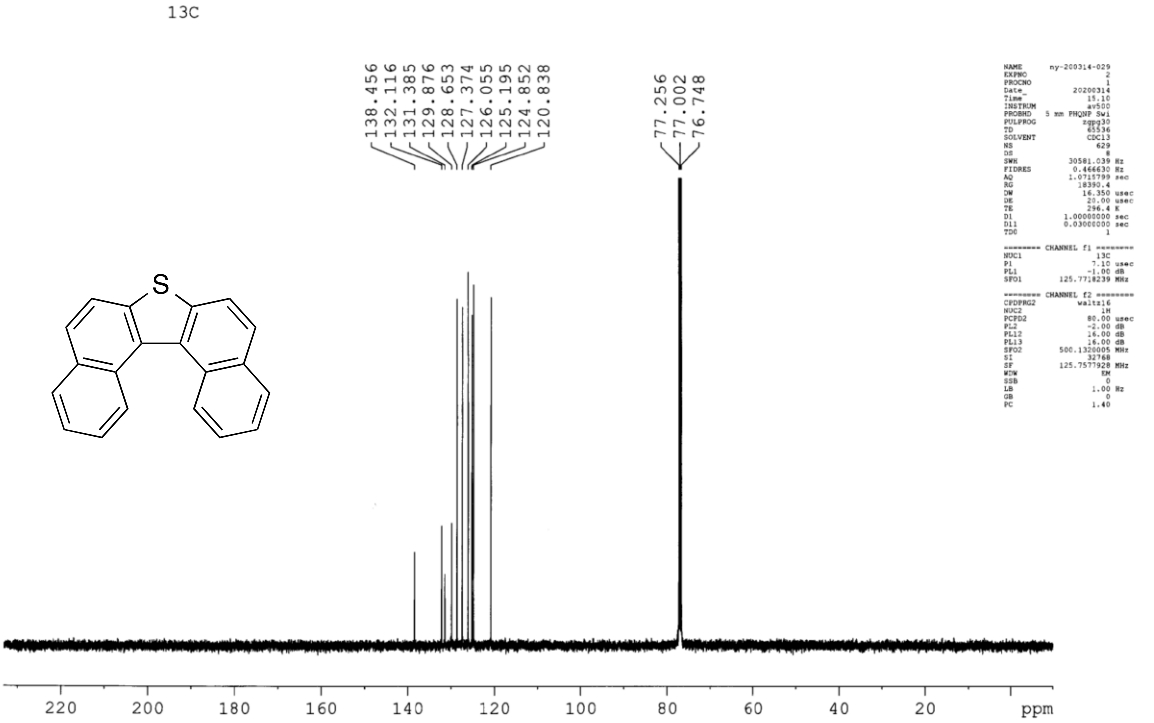

7-(4-Methylbenzenesulfonyl)-7*H*-dibenzo[*c*,*g*]carbazole (12c)

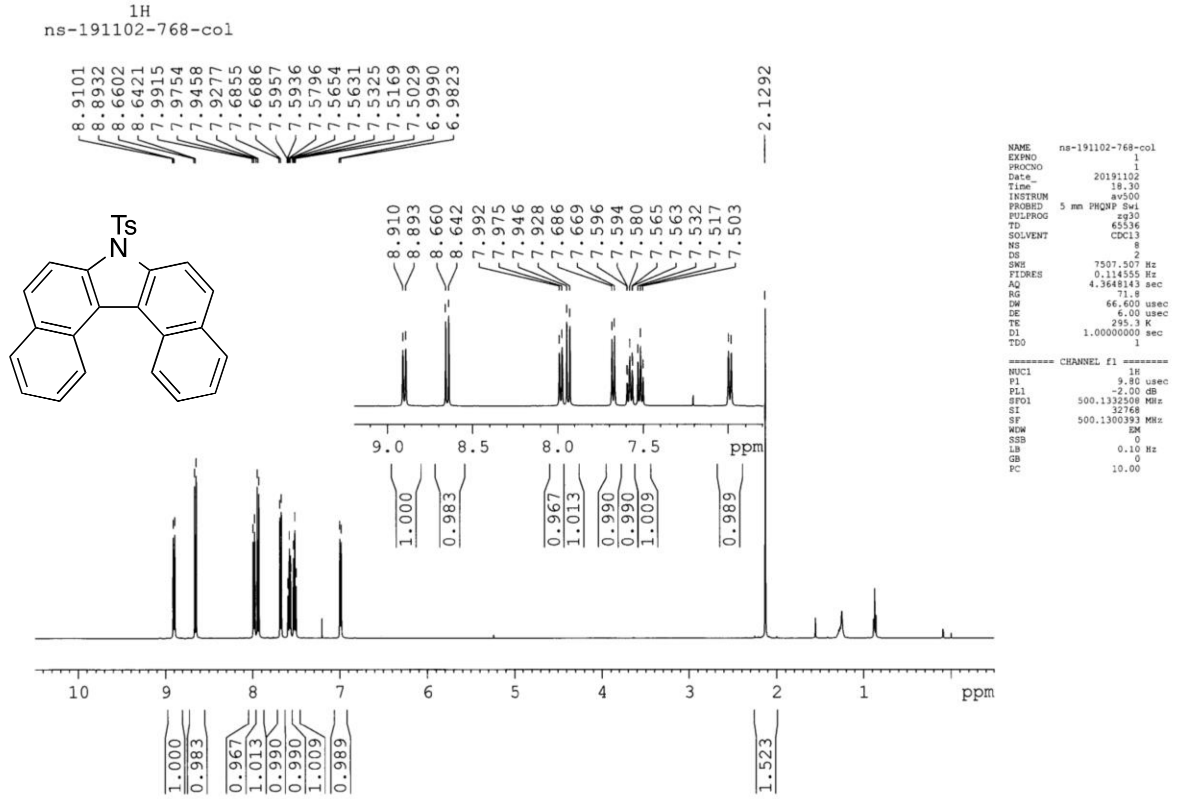

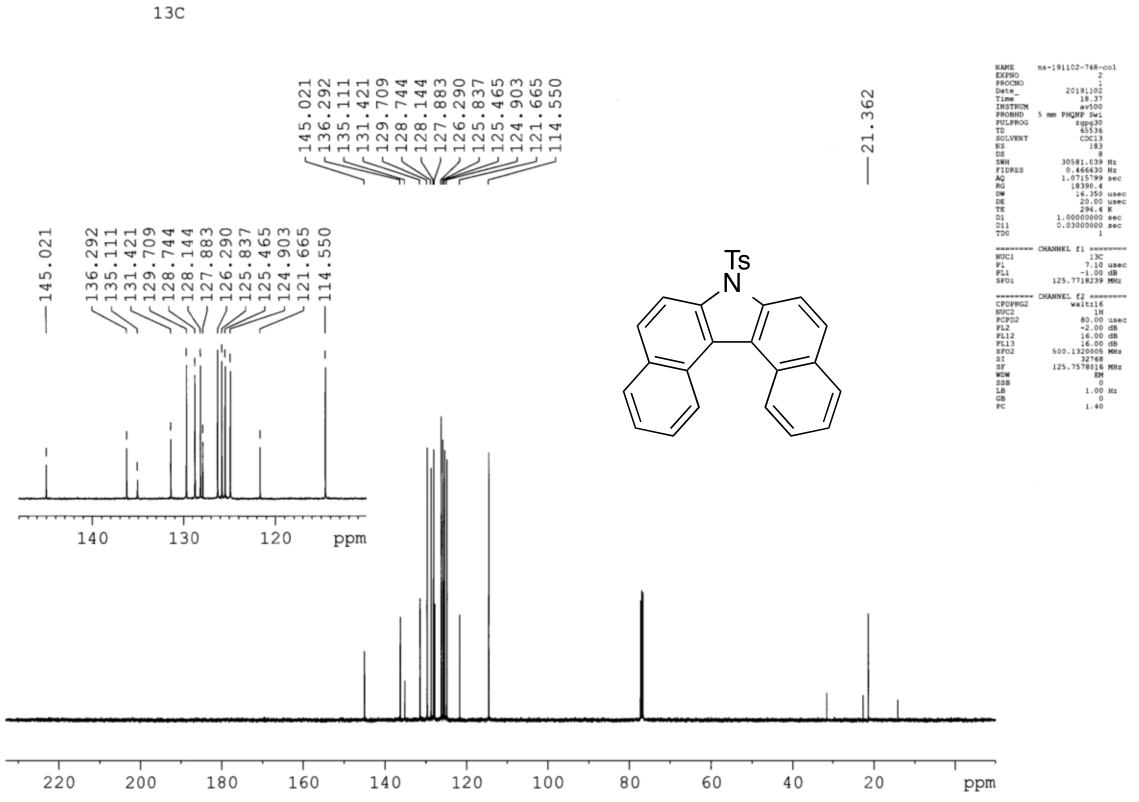

Benzo[*b*]benzo[5,6]phenanthro[4,3-*d*]thiophene (15)

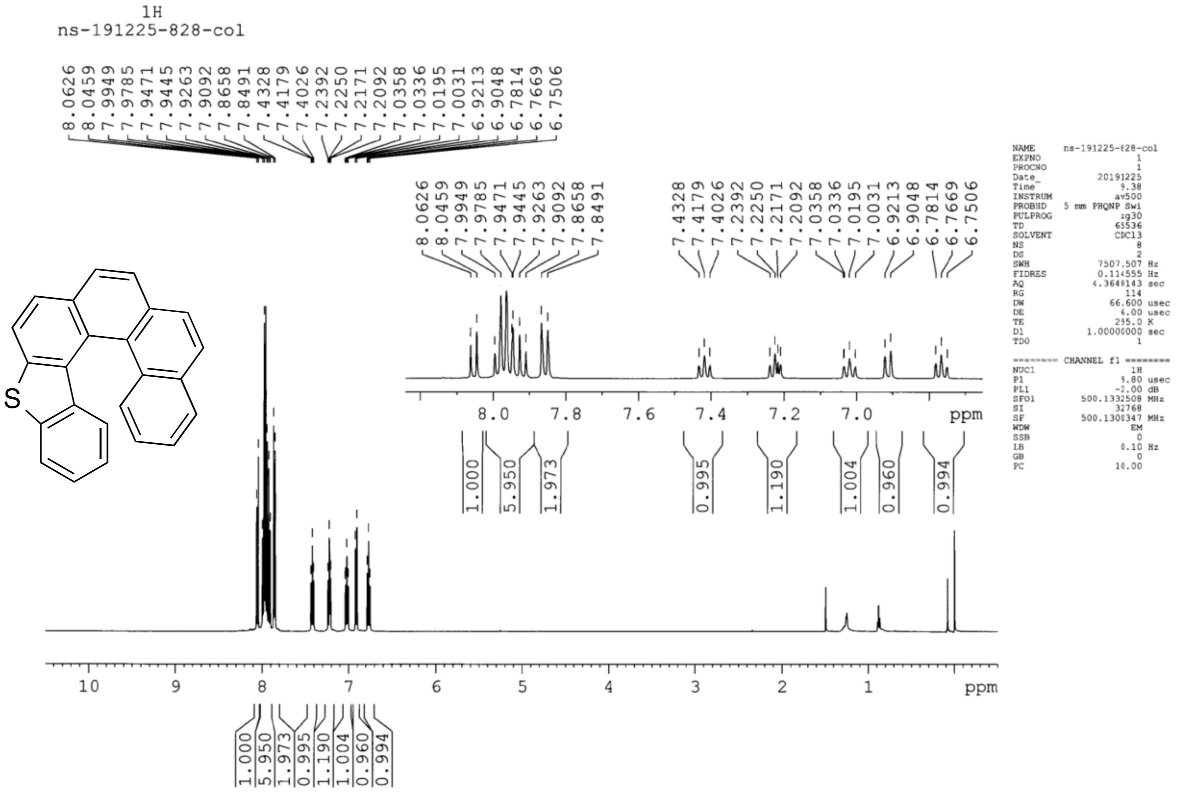

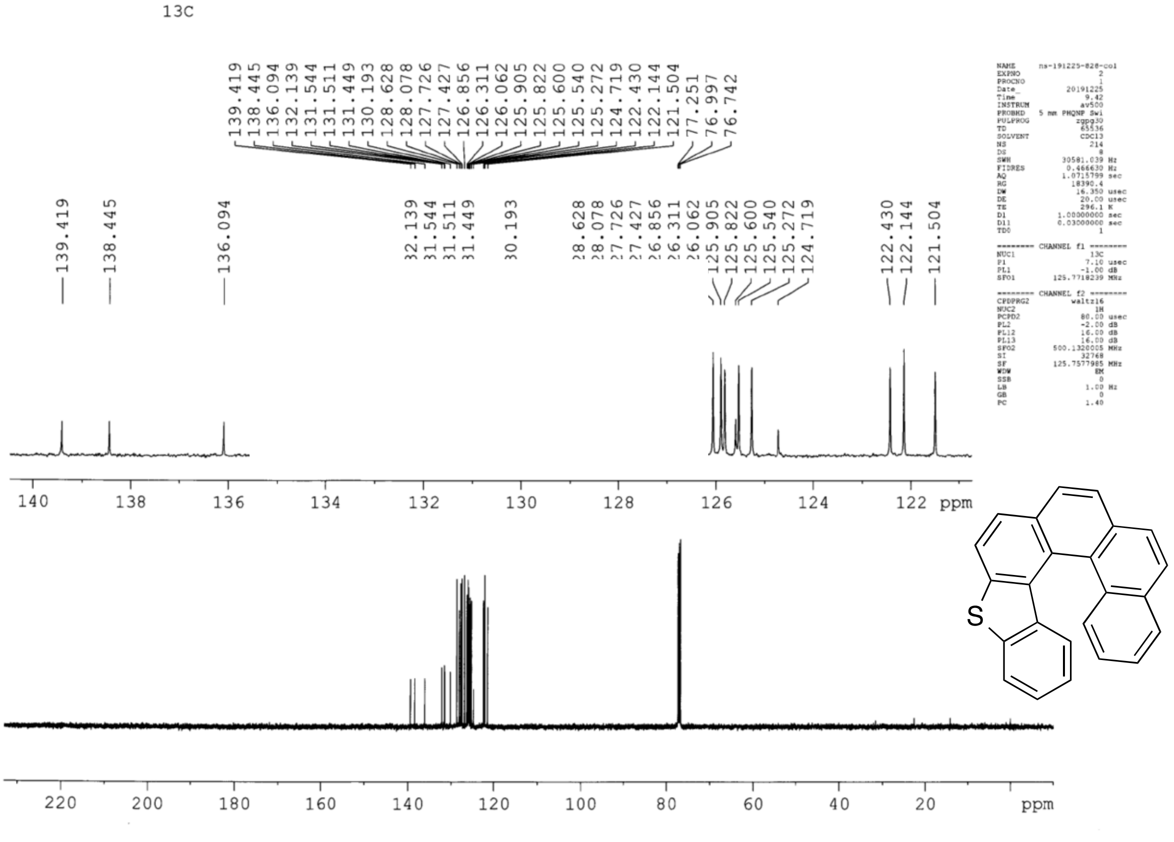

Supplement: File 1 — Detailed experimental procedures and spectral data. [file Beilstein_J_Org_Chem-17-396-s001.pdf]
